# Emerging zoonotic diseases originating in mammals: a systematic review of effects of anthropogenic land‐use change

**DOI:** 10.1111/mam.12201

**Published:** 2020-06-02

**Authors:** Rebekah J. White, Orly Razgour

**Affiliations:** ^1^ Biosciences University of Exeter Living Systems Institute Exeter EX4 4QD UK; ^2^ Biological Sciences University of Southampton Life Sciences Building, Highfield Campus Southampton SO17 1BJ UK; ^3^ Biosciences University of Exeter Hatherly Laboratories Exeter EX4 4PS UK

**Keywords:** anthropogenic land‐use change, deforestation, epidemic, global change, mammals, urbanisation, zoonotic diseases

## Abstract

1. Zoonotic pathogens and parasites that are transmitted from vertebrates to humans are a major public health risk with high associated global economic costs. The spread of these pathogens and risk of transmission accelerate with recent anthropogenic land‐use changes (LUC) such as deforestation, urbanisation, and agricultural intensification, factors that are expected to increase in the future due to human population expansion and increasing demand for resources.
2. We systematically review the literature on anthropogenic LUC and zoonotic diseases, highlighting the most prominent mammalian reservoirs and pathogens, and identifying avenues for future research.
3. The majority of studies were global reviews that did not focus on specific taxa. South America and Asia were the most‐studied regions, while the most‐studied LUC was urbanisation. Livestock were studied more within the context of agricultural intensification, carnivores with urbanisation and helminths, bats with deforestation and viruses, and primates with habitat fragmentation and protozoa.
4. Research into specific animal reservoirs has improved our understanding of how the spread of zoonotic diseases is affected by LUC. The behaviour of hosts can be altered when their habitats are changed, impacting the pathogens they carry and the probability of disease spreading to humans. Understanding this has enabled the identification of factors that alter the risk of emergence (such as virulence, pathogen diversity, and ease of transmission). Yet, many pathogens and impacts of LUC other than urbanisation have been understudied.
5. Predicting how zoonotic diseases emerge and spread in response to anthropogenic LUC requires more empirical and data synthesis studies that link host ecology and responses with pathogen ecology and disease spread. The link between anthropogenic impacts on the natural environment and the recent COVID‐19 pandemic highlights the urgent need to understand how anthropogenic LUC affects the risk of spillover to humans and spread of zoonotic diseases originating in mammals.

## Graphical abstract

Identifying gaps in our understanding of the effects of anthropogenic land‐use change on the spread of zoonotic pathogens originating in mammals. We found skew in the geographic distribution of studies, with Africa being under‐represented, key hosts of zoonotic diseases, like bats, being under‐studied, key pathogen groups not studied in all hosts, and impacts of important land‐use change drivers, such as agricultural intensification, being under‐studied beyond livestock.

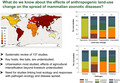

## Introduction

Three quarters of emerging human pathogens are zoonotic, that is they are transmitted from other vertebrate animals to humans (Taylor et al. 2001). Zoonoses have a considerable ecological and socio‐economic impact, as well as being a burden on global economies (Cascio et al. 2011). Emerging infectious diseases (EIDs) are newly recognised or reappearing diseases that have been detected in a population for the first time and are rapidly increasing in prevalence or geographic range (Lederberg et al. 1992). Zoonoses account for nearly two thirds of EIDs, and the majority of zoonoses originate in wild animals (Jones et al. 2008). For new emergences, it is important to identify the source of the outbreak and the epidemiological factors that allow it to spread, but many methods for collecting these data are still under development (DiEuliis et al. 2016). A major scientific challenge in EID research is developing realistic and cost‐effective ways to predict, prevent, and respond to outbreaks (Lendak et al. 2017).

The advancement of diseases has been described as "a side effect of the growth of civilisation" (Dobson & Carper 1996), and zoonoses are no exception. Recent unprecedented rates of anthropogenic land‐use change (LUC), including urbanisation, agricultural conversion or intensification, deforestation, and habitat fragmentation, have lead to run‐away loss of natural environments to human development. LUCs that alter the local environment and human–wildlife interactions can be a prominent source of zoonotic diseases because they remove or reduce the natural habitats and home ranges of many species, forcing them to live in closer proximity to humans. This becomes an issue if the species is a host for a zoonotic disease (Jones et al. 2013). Pathogen transmission tends to increase in response to anthropogenic change, but this effect is not universal (Gottdenker et al. 2014).

Although not all zoonotic pathogens are strongly associated with particular types of non‐human hosts (Woolhouse & Gowtage‐Sequeria 2005), the interactions at the host–pathogen interface are still important for understanding how a disease may spread if populations are affected by anthropogenic LUC. Therefore, it is important to consider the differences between taxa of zoonotic pathogens and hosts, because changes in the dynamics of the interface may be partly responsible for disease emergence (Ko et al. 2009). Mammals are particularly important hosts of zoonotic EIDs. High mammal species richness in combination with anthropogenic LUC in forested tropical areas has been identified as a key predictor of risk of zoonotic disease emergence (Allen et al. 2017). Hence, this review addresses the effects of anthropogenic LUC on the spread of zoonotic diseases, focusing on analysing trends in the literature, identifying key mammalian reservoirs and pathogen taxa, assessing emerging threats, and highlighting avenues for future research.

## Methods

Search terms for the systematic review were identified through pilot searches of ‘Web of Science’, with initial keywords and phrases ‘land‐use change’, ‘zoonotic diseases’, and ‘emergence’ to gain an overview of important topics covered in the literature. From this, the most important anthropogenic LUCs identified were urbanisation, deforestation, habitat fragmentation, and agricultural intensification, leading to the following search pattern carried out in ‘Web of Science’ for the years 1970–2019: TOPIC: (*zoonotic diseases OR zoonoses OR rodent‐borne diseases OR bat‐borne diseases*) AND TOPIC: (*anthropogenic land‐use change OR anthropogenic land cover change OR deforestation OR urbanisation OR urbanization OR agricultural intensification OR agriculture expansion OR agriculture conversion OR urban expansion OR urban sprawl OR land conversion OR fragmentation*). The final search was carried out in October 2019.

Of the 357 papers recovered from the final search (Appendix S1), 276 were retained following application of the Preferred Reporting Items for Systematic Reviews and Meta‐Analyses (PRISMA; Moher et al. 2009). The decision to reject papers was independently revised by two people. Initially, seven duplicate papers, book chapters, and conference abstracts were omitted. After reading the abstracts of the remaining papers, 74 papers were removed because they did not directly study either anthropogenic LUC (58 papers) or zoonotic diseases (15). These include studies that only mentioned LUC or zoonotic diseases as a potential future problem. Trends in the literature were analysed in the 276 papers that were retained, focusing primarily on a subset of 136 papers that specifically studied mammalian hosts. The following parameters were recorded: publication year, study region(s), study type (review, modelling, and empirical, including observation and experimental studies), host taxa, LUC, pathogen type, and whether or not it was a study of vector‐borne disease (in which a vector, such as an insect or tick, transmits the pathogen between hosts; Appendix S2). Chi‐square tests were used to identify associations between the different parameters (five tests), and *P* values were adjusted for multiple testing.

## Results and Discussion

### General trends in the literature

**Fig. 1:**
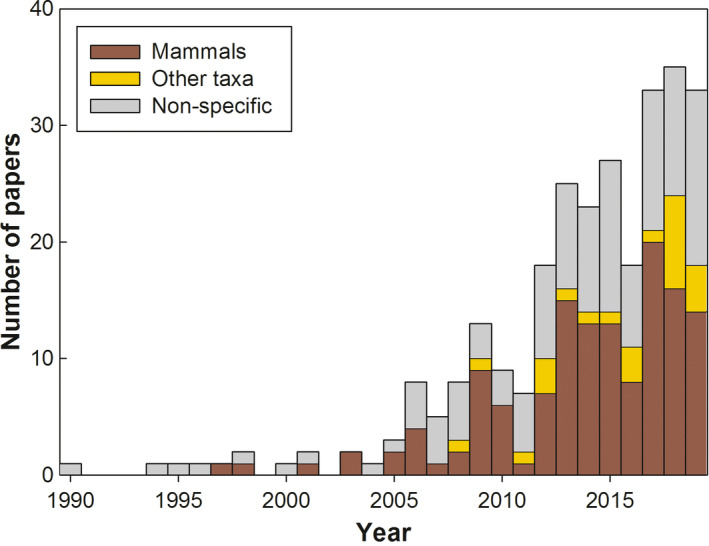
Increase in the number of publications addressing the effect of anthropogenic land‐use change on the spread of zoonotic diseases with time (year of publication), divided into mammal, other taxa (birds, arthropods, and amphibians), and non‐specific papers. [Colour figure can be viewed at wileyonlinelibrary.com]

We compiled a total of 276 studies on zoonotic diseases and anthropogenic LUC, published between 1990 and 2019 (Appendix S2). Of the 276 studies included in the first step of this review, nearly half (136 studies; 49%) were focused on mammals, while 42% were not focused on a specific host taxon. The remaining 9% of studies either were focused on or included birds (12 studies), arthropods (12 studies), or frogs (one study). The first four studies, published 1990–1996, were review papers that did not focus on specific host taxa. The first mammal paper was published in 1997 and was an empirical study (Pavlovic et al. 1997). Similar to Gottdenker et al. (2014), we found a trend of increased rates of publication with time, which continued in the last seven years. Overall rate of publications increased in 2006 from 1 to 3 papers to > 5 papers, in 2012 to > 18 papers, and in 2017 to > 33 papers per year. Mammal papers followed a similar trend, with the exception of a dip in publications in 2007 and 2011 (Fig. 1). The two major points of increase in publication rates (2012 and 2017) appeared to follow periods of discovery or outbreaks of major zoonotic diseases, such as the discovery of Middle East respiratory syndrome coronavirus in Saudi Arabia (2012) and the major Ebola outbreak in Africa (2014–2016; WHO 2019). However, the increase in number of publications may simply reflect the general increase in scientific publications over that period. Furthermore, like any comparative study, this systematic search may have missed pertinent papers; therefore, results should be considered as a sample of the broader literature.

**Fig. 2:**
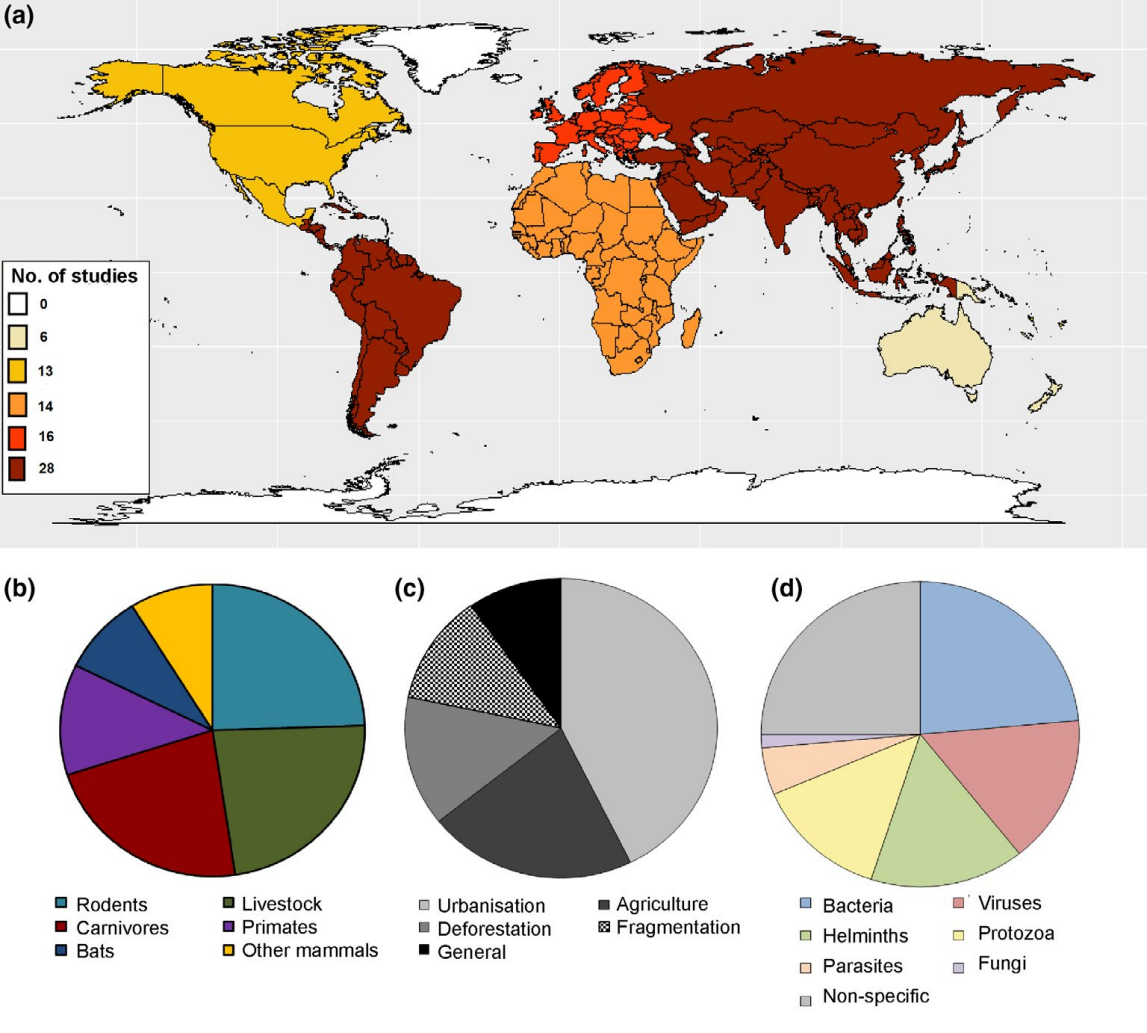
Trends in the publications on anthropogenic land‐use change and mammalian zoonotic diseases: colour‐coded numbers of papers per key geographic region (a), overall proportion of papers including different host taxa (b), land‐use change categories (c), and pathogens (d). [Colour figure can be viewed at wileyonlinelibrary.com]

Nearly a quarter of studies of mammals were global (24%, 33 studies), 20% were carried out in each of South America and Asia, and around 10% in each of Europe, Africa, and North America (Fig. 2a). Significant associations were identified between mammalian hosts and geographic region, whereby carnivores were studied more in Europe, primates in Africa, rodents in North America, and livestock globally (χ^2^ = 80.08, d.f. = 25, *P* < 0.001). Of the LUCs, urbanisation was studied more in Europe and agricultural intensification globally (χ^2^ = 50.1, d.f. = 15, *P* < 0.001).

**Fig. 3:**
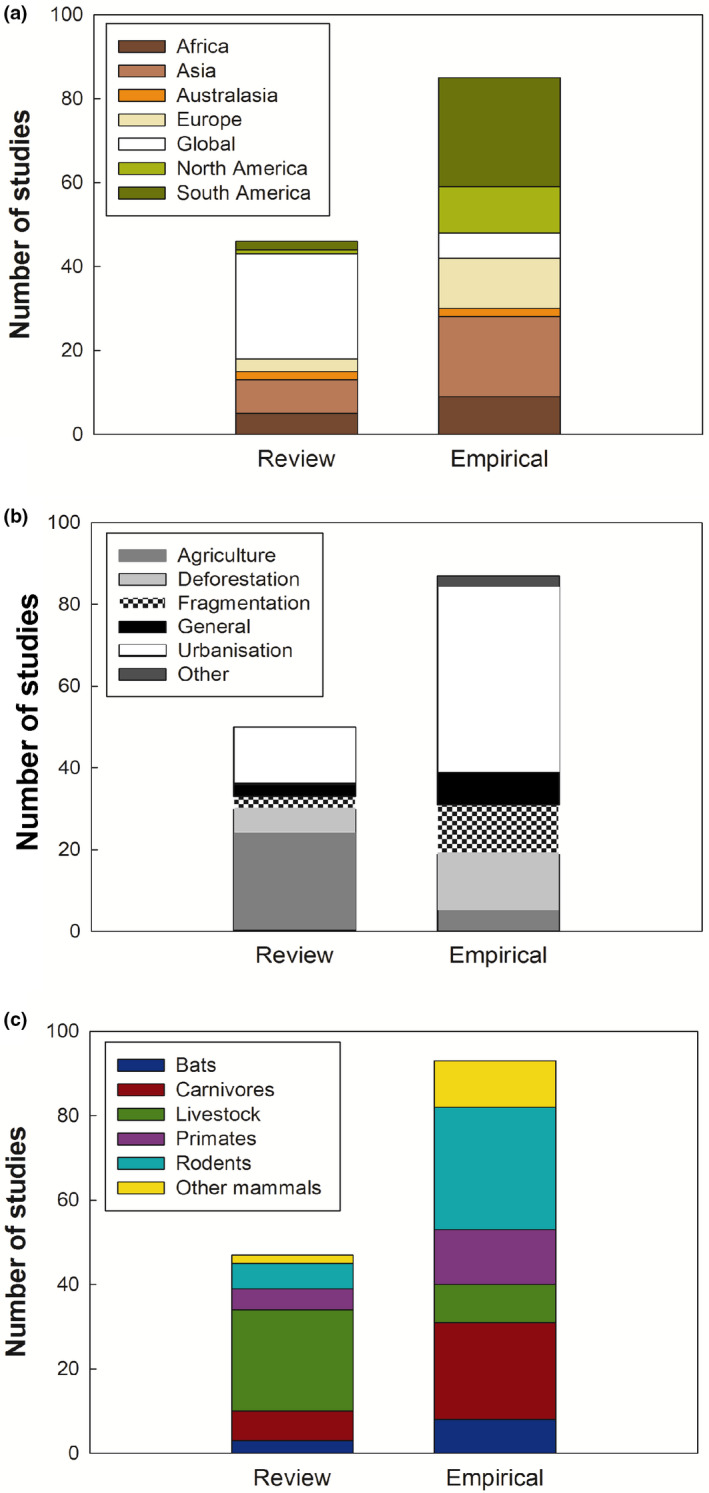
Number of review papers and empirical studies in the mammalian dataset divided according to geographic regions (a), anthropogenic land‐use change categories (b), and mammalian hosts (c). [Colour figure can be viewed at wileyonlinelibrary.com]

The majority of non‐host specific studies were reviews (72%, 84 studies), while the majority of studies of mammals were empirical (63%, 85 studies). Only 4% of overall studies used modelling approaches (5% of studies of mammals). In the mammal dataset, empirical studies were mainly carried out in South America (31%) and Asia (22%), studied rodents (34%), and focused on urbanisation (53%). In contrast, review studies were mostly global (57%), studied livestock (54%), and focused on agricultural intensification (54%; Fig. 3).

**Fig. 4:**
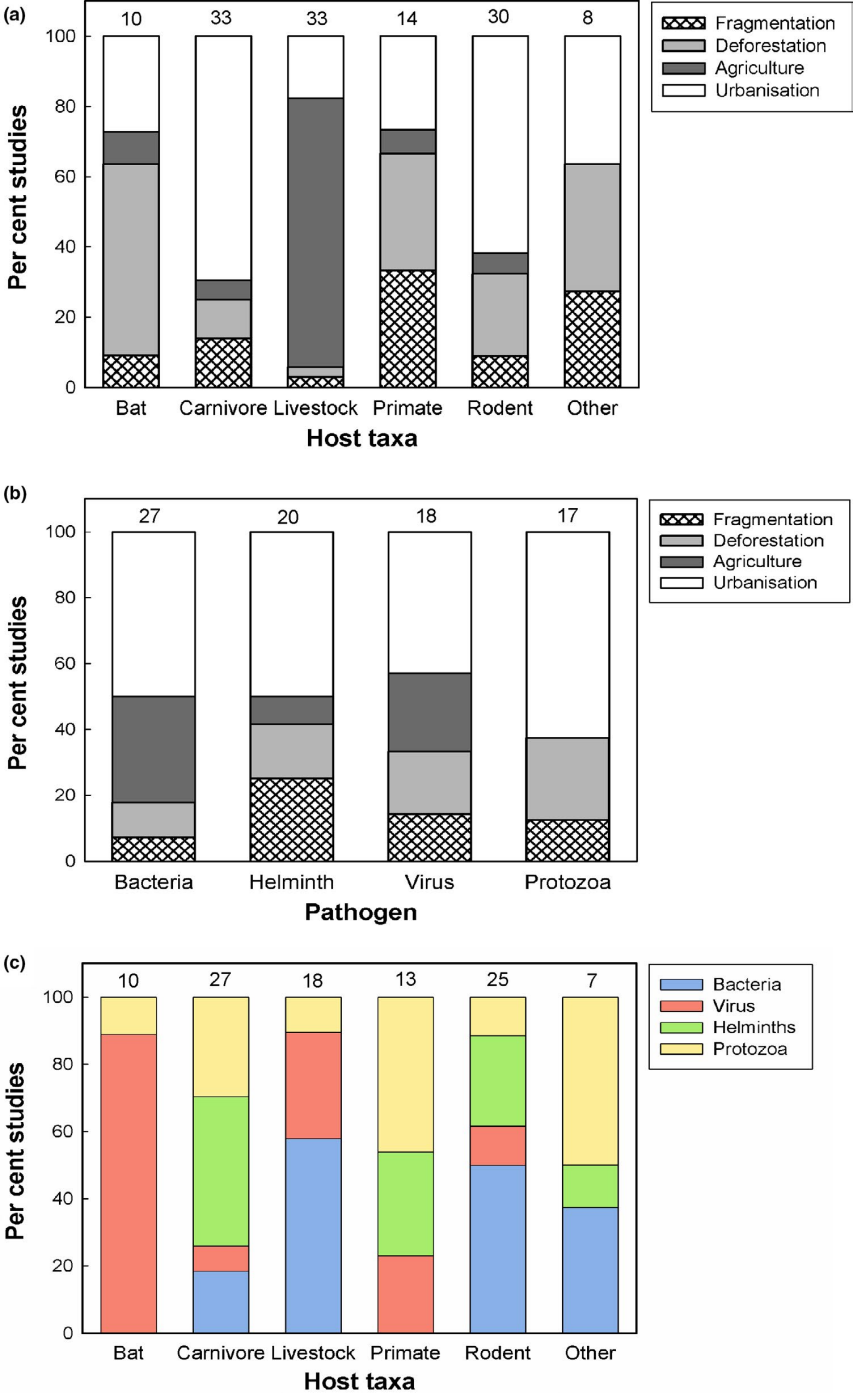
Associations between mammalian host taxa and land‐use change categories (a), pathogens and land‐use change categories (b), and mammalian host taxa and pathogens (c), based on the per cent of published studies covering each category. The number of studies included in each category is shown above each bar. [Colour figure can be viewed at wileyonlinelibrary.com]

Significant associations were identified between LUC categories and mammalian host taxa (χ^2^ = 98.02, d.f. = 15, *P* < 0.001; Fig. 4a). Primarily, livestock were studied more within the context of agricultural intensification, but less with urbanisation, while carnivores were studied more with urbanisation, bats with deforestation, and primates with habitat fragmentation. Pathogen taxa were not associated with LUC categories (χ^2^ = 12.55, d.f. = 9, *P* > 0.05; Fig. 4b). However, we did find associations between pathogens and mammalian hosts (χ^2^ = 63.88, d.f. = 15, *P* < 0.001; Fig. 4c), whereby bats were studied more with viruses, carnivores with helminths, and primates with protozoa.

### Most‐studied hosts of zoonotic pathogens under land‐use change

Different animal hosts can have life‐history traits or life cycles that impact disease spread and determine whether the pathogen can overcome the species barrier. It is important to understand both sides of the host–pathogen interface in order to be able to predict spillover to humans, amplification, and spread of zoonotic diseases (Johnson et al. 2015). The most frequently studied mammalian taxon was rodents (36 studies; 27%), closely followed by livestock (34; 25%) and carnivores (33; 24%). The remaining studies focused on non‐human primates (18; 13%), bats (13; 10%), and other wild mammals (13; 10%; Fig. 2b). Eleven studies covered more than one mammalian group.

#### Rodents

Rodents are important reservoirs of emerging zoonotic viruses because they come into close contact with livestock in the agricultural setting and humans in urban areas (Luis et al. 2013). We found that the main LUC covered in the rodent studies was urbanisation (21 studies; 58%), followed by deforestation (8; 22%). Only two studies covered the impacts of agricultural intensification and three covered habitat fragmentation (Fig. 4a). The main pathogens studied were bacteria (13 studies; 36%) and helminths (7; 19%). Viruses and protozoa were included in three studies each, and fungi and parasites in one. A quarter of rodent studies did not focus on a specific pathogen (Fig. 4c).

Rats have been reported to harbour an expansive range of zoonoses in both developing and developed countries, such as bartonella in *Rattus norvegicus* in Canada (Rothenburger et al. 2018), *Leptospira* spp. in Malaysian Borneo (Blasdell et al. 2019), and helminths in Argentina (Hancke & Suarez 2018). Furthermore, *Yersinia pestis* (black plague) circulates at low levels in rodent populations. Deforestation and urbanisation increase the risk of re‐emergence of this disease in humans, because these LUCs can result in the emergence of new vectors, expansion of rodent habitats, and modification of population dynamics (Duplantier et al. 2005). Recent studies considering diseases associated with non‐*Rattus* rodents include intestinal helminths in Japanese field mice *Apodemus speciosus* (Anders et al. 2019), infections from *Nosopsyllus fasciatus* ticks in mice and voles in Berlin (Maaz et al. 2018), and cutaneous leishmaniasis in the fat sand rat *Psammomys obesus,* and the Libyan jird *Meriones libycus,* in Saudi Arabia (Abuzaid et al. 2017).

#### Livestock

Livestock are prevalent zoonotic reservoirs. LUC factors promoting transmission are usually associated with farming conditions and practices and their demographic consequences (Tomley & Shirley 2009). Of the 34 livestock studies reviewed, 77% covered the impacts of agricultural intensification, and six studies (18%) covered urbanisation (Fig. 4a). The main pathogens covered in livestock studies were bacteria (10 studies; 29%) and viruses (5; 15%); 44% of studies did not cover a specific pathogen. Other pathogens covered were protozoa and parasites (1 study; Fig. 4c).

Bovine leptospirosis was found in 13% of dairy cows in urban and peri‐urban Tajikistan, including in areas where large numbers of human and animals co‐exist (Rajala et al. 2017). Brucellosis is another emerging threat from cattle, particularly in Africa, and is a prominent issue for developing economies (Ducrotoy et al. 2014). Viral diseases from livestock also pose threats in the developing world, with increased risk of infection potentially associated with deforestation (Bayry 2013). Clark and Soares Magalhães (2018) show that the prevalence of Q fever, caused by a bacterium that attaches to dust and is spread by sheep or goats, is partly associated with urbanisation level and stocking density, which increases greatly when agriculture is intensified. There are many zoonoses associated with pigs in Asia, including leptospirosis, Trichinella, and hepatitis E virus, and the lack of epidemiological studies into these diseases may allow spread to increase as agriculture becomes more intensive (Okello et al. 2015). Reducing global consumption of animal‐based food products is a way to reduce zoonotic disease spread associated with agriculture, as there would be reduced reliance on agricultural intensification. The literature covers the main livestock zoonoses identified by the UK government (HSE 2019); however, epidemiology and surveillance studies of rarer diseases, such as *Erysipeloid* bacterial infection and anthrax, are still lacking.

#### Carnivores

The majority of the 33 carnivore studies included in our review (76%) were focused on impacts of urbanisation. Impacts of deforestation and habitat fragmentation were covered in four studies each, while agricultural intensification featured in only two studies (Fig. 4a). The main pathogens covered in carnivore studies were helminths (12 studies; 36%) and protozoa, primarily *Leishmania* (8; 24%). Bacteria and parasites were covered in three studies each (Fig. 4c). Despite the role of carnivores in transmission of rabies to humans (Abera et al. 2015), only a single study covered viruses.

Zoonotic diseases associated with canines emerge in urbanised areas across the world, caused by, for example, *Echinococcus multilocularis* in medium‐sized cities in France (Umhang et al. 2014), *Brucella canis* in urban Argentina (Marzetti et al. 2013), and vector‐borne *Ehrlichia* spp. and *Babesia* spp. in Costa Rica (Springer et al. 2019). Dingoes in urban Australia are associated with the transmission of parasitic zoonoses, as they can reach higher population densities in urban areas than in their natural habitats (Mackenstedt et al. 2015). Domestic dogs are prominent reservoirs for visceral leishmaniasis, a disease caused by an obligate intracellular protozoan parasite (de Oliveira et al. 2015). A study investigating leishmaniasis in dogs and other wild mammals in protected areas in Brazil found evidence of outbreak foci becoming established following environmental modifications (Donalisio et al. 2017). Other carnivores associated with zoonotic diseases include genets with *Bartonella* spp. and *Coxiella burnetii* in Spain (Millán et al. 2016), wild and domestic carnivores and Brazilian spotted fever in fragmented forests in São Paulo (Scinachi et al. 2017), and giant pandas and hookworm in urbanised areas in China (Xie et al. 2017). Although some of the studies were focused on parasitic nematodes in specific locations, the immense impact of human activities and politics on zoonotic helminths in carnivores around the globe (Otranto & Deplazes 2019) is under‐represented in the literature.

#### Non‐human primates

Of the 18 non‐human primate studies reviewed, five each looked at impacts of deforestation and habitat fragmentation (27% each) and four at urbanisation (22%; Fig. 4a). The main pathogens covered were protozoa (6 studies; 33%), helminths (4; 22%), and viruses (3; 17%; Fig. 4c). Several viral and parasitic infections can naturally transmit between non‐human primates and humans (Parker et al. 2007, Salyer et al. 2012). While parasites of macaque monkeys *Macaca* sp. are a prominent issue, only three papers cover this. Some species have been found to have a higher prevalence of plasmodium hosting as a result of forest fragmentation, including disturbed forest areas and forest edges (Moyes et al. 2016), and habitat fragmentation increases the prevalence of *Oesophagostomum* and *Trichuris* helminth eggs in suburban areas in Japan (Arizono et al. 2012). Other primates associated with LUC and parasites are vervet and proboscis monkeys *Chlorocebus pygerythrus* and *Nasalis larvatus* (Klaus et al. 2017, Thatcher et al. 2018). Viruses spreading in response to LUC were not associated with a particular group of primates, and only dengue fever was specifically covered in the studies (Twiddy et al. 2003, Rey et al. 2010). However, other viruses, including pox, Marburg, and Ebola, can be indirectly transmissible to humans via insect vectors or rodents (Taku et al. 2007), highlighting another gap in the literature.

#### Bats

Although bats are regarded as important hosts of zoonotic pathogens (e.g. Allocati et al. 2016), only 13 studies so far have addressed the effects of anthropogenic LUC on zoonotic diseases emerging from bats. These studies have mainly been focused on the impacts of deforestation (6 studies; 46%) and to a lesser extent urbanisation (3; 23%; Fig. 4a), and primarily covered viruses (8; 62%). Other pathogens included were protozoa, parasites, and fungi, with one study each (Fig. 4c).

Bats have diverse and unique life‐history traits that allow the spread of pathogens, including the ability to migrate long distances and their tendency to aggregate in crowded roosts, which facilitates both intraspecific transmission and interspecific transmission of microbes (Hayman et al. 2012, Luis et al. 2015). Due to their long lifespan and adapted intracellular processes that enable survival of some types of infection (Brook & Dobson 2015), bats are natural reservoir hosts of over 200 viruses, bacteria, and fungi (Allocati et al. 2016). Prevalence of pathogens can be affected by LUC, for example mucocutaneous leishmaniasis and parasites by urbanisation (Shapiro et al. 2013, Nunes et al. 2017) and henipaviruses by deforestation (Field 2009, Pernet et al. 2014). The risk of henipaviruses emerging from Old World fruit bats increases due to anthropogenic forest disturbance, which results in changes to resource provisioning and behaviour of these hosts (Kessler et al. 2018).

Emergences of bat‐borne viruses are challenging to predict in an environment that has been subject to extensive LUC. The high number of pathogens associated with bats, especially bacteria, has not been represented in the reviewed literature, indicating that further studies are required to allow a comprehensive understanding of bats and zoonoses under LUC.

#### Other mammalian hosts

Of the 136 studies of mammals, 13 covered wild mammals other than rodents, carnivores, primates, and bats. Changes in zoonotic bacteria in deer reservoirs have been associated with LUC, such as Lyme disease, *Ehrlichia chaggeensis,* and *Anaplasma phagocytophilum* (Manangan et al. 2007, Millins et al. 2017). Furthermore, grey seals *Halichoerus grypus,* have been associated with *Campylobacter* in Europe (Baily et al. 2015), and pika *Ochotona princeps,* with *Echinococcus multilocularis* in Asia (Marston et al. 2014a).

### Studies of vector‐borne disease

The full dataset included 47 studies of vector‐borne disease, 45% of which did not focus on a specific host, while 28% (13 studies) covered mammals. The studies of mammalian disease covered the impacts of urbanisation (5 studies; 39%) and bacterial pathogens (6; 46%). Swei et al. (2020) found that the majority of emerging vector‐borne zoonoses are transmitted by ticks and mosquitoes, and that the most common pathogens are *Rickettsiaceae* bacteria and RNA viruses. Although the driver for an emergence is not always known, these studies indicate LUC is likely to play an important role.

### Most‐studied pathogens and parasites under land‐use change

Understanding how the type of pathogen affects the epidemiology of the zoonotic disease is necessary for the development of treatments and prediction of outbreaks (Morse et al. 2012). To become zoonotic, a pathogen must overcome a hierarchal series of barriers (Plowright et al. 2017) and eventually be able to adapt successfully to fluctuating environments posed by the human immune response (Regoes et al. 2012). It must acquire new characteristics to overcome host species barriers, thus transmitting to and between humans.

The most common pathogens and parasites studied within the context of anthropogenic LUC and mammalian hosts are bacteria (33 studies; 24%), viruses (22; 16%), helminths (22; 16%), and protozoa (20; 15%). A quarter of mammalian studies reviewed (35 studies) did not focus on a specific pathogen (Fig. 2d). While studies of bacteria were distributed relatively evenly across the globe, studies of protozoa were more common in South America (50%) and studies of helminths and viruses in Asia (36% and 27%, respectively). Bacterial pathogens were most commonly studied in rodents (13 studies; 39%) and livestock (11; 33%), viruses in bats (8; 36%), and helminths and protozoa in carnivores (55% and 40% respectively; Fig. 4c). We identified four emerging zoonoses associated with anthropogenic LUC that have received most research attention thus far, possibly due to global concern or public health impacts: the bacteria *Leptospira* causing leptospirosis and *Bartonella* causing bartonellosis, the parasitic tapeworm *Echinococcus*, and the intracellular protozoa *Leishmania* causing leishmaniasis.

#### Bacteria

All types of anthropogenic LUCs considered here (i.e. urbanisation, agricultural conversion/intensification, deforestation, and fragmentation) have the potential to increase the risk of an emergence of a bacterial zoonotic disease across the globe. For example, the relative abundance of *Bartonella* in rodents has been correlated with the increasing level of land disturbance and deforestation in Peru (Cortez et al. 2018), risk of brucellosis increases with agricultural intensification (Ducrotoy et al. 2014), and the incidence of *Borrellia* spp. in mammals increases with habitat fragmentation across Europe (Millins et al. 2018). *Borrellia* spp., the cause of Lyme disease, was only studied in three papers, despite its known association with changes in land‐use patterns (CDC 2019). *Leptospira*, hosted by rodents and livestock, causes leptospirosis in humans and spreads particularly well in tropical regions (Levett 2001). It has been identified as a worrying emerging zoonotic disease associated with urbanisation (Rajala et al. 2017, Blasdell et al. 2019).

Antibiotic resistance in livestock continues to be a pressing issue worldwide, for example the emergence of methicillin‐resistant *Staphylococcus aureus* (MRSA; Mehndiratta & Bhalla 2014). Antibiotics need to be used judiciously (Tilman et al. 2002) to reduce the risk of rapid outbreaks of antibiotic‐resistant zoonotic EIDs that could spread extremely quickly. Despite this potentially major global issue, the link between antibiotic resistance and zoonotic disease emergence due to agricultural intensification is not well reported in the literature.

#### Viruses

Viruses can generate *de novo* diversity over a short period due to their ability to mutate rapidly (Duffy et al. 2008). Despite recent technological developments allowing the discovery of novel zoonotic viruses (Marston et al. 2014b), our understanding of how a zoonotic virus emerges and spreads is still incomplete. RNA viruses are particularly likely to emerge as they can adapt quickly to new environmental pressures through rapid replication times and mutation rates (Domingo & Holland 1997). Their risk of emergence can increase under LUC, for example Ross River virus under agricultural intensification (Carver et al. 2009) and SARS coronavirus from bats under several LUCs (Field 2009). However, surveillance is poor, with only a few studies focused on high‐risk environments, such as tropical countries. Numerous zoonotic viruses are emerging alongside agricultural intensification in the developing world (Bayry 2013). While some are considered in association with specific hosts, such as Nipah virus with bats and Ross River virus with sheep (Carver et al. 2009, Pulliam et al. 2012), the number of studies in this area is not sufficient to understand the range of potential outbreaks, such as influenza, Hendra, Newcastle disease virus, and more (Bayry 2013).

#### Helminths

Helminths are parasitic worms that are usually transmitted via food or faeces. Infection is reported all around the world, with a number of papers focusing on urbanisation impacting *Echinococcus multilocularis* spread by carnivores, for example foxes *Vulpes vulpes* in Switzerland (Otero‐Abad et al. 2017) and dogs in France (Umhang et al. 2014). A change in landscape dynamics resulting from deforestation was found to affect disease distribution of human alveolar echinococcosis following changes in rodent host distribution (Giraudoux et al. 2003). The strong link between LUC and rodent and carnivore‐borne *Echinococcus* infection highlights the need for improved mitigation techniques. In South‐East Asia, helminth sharing among rodents becomes harder to contain under conditions of habitat fragmentation due to a less connected and more modular rodent–helminth network (Bordes et al. 2015). Only a few of the studies reviewed were focused on the spread of helminths in South America, where stray dogs often roam freely in urban areas and are known for spreading other zoonotic pathogens, suggesting there may be unreported helminth outbreaks.

#### Protozoa

Instances of zoonotic infections caused by protozoa (obligate intracellular parasites) have been linked with many types of anthropogenic LUC. Giardiasis is a diarrhoeal disease caused by *Giardia* spp. in the gastrointestinal tract. It has been described as re‐emerging, has multiple hosts, and transmission can occur when contact is made with excrement (Thompson 2000). Deforestation has been associated with outbreaks of *Giardia* spp. from many hosts, including livestock, *Coendou villosus*, *Oligoryzomys* sp., *Didelphis aurita,* and *Marmosops incanus* (Lallo et al. 2009).

*Leishmania*, the protozoan causing leishmaniasis, is hosted by bats and rodents (cutaneous; Shapiro et al. 2013, Abuzaid et al. 2017) or canines (visceral; de Oliveira et al. 2015). Prevalence of visceral vector‐borne leishmaniasis was found to increase with urbanisation in Brazil (de Oliveira et al. 2015). Visceral, as opposed to cutaneous infections, can severely affect several organs in humans. The severity of this disease means that surveillance needs to continue as urbanisation increases, to prevent this from becoming a neglected zoonotic disease. An increased risk of infection by plasmodium, the malaria parasite, has been associated with disturbed forests and the presence of non‐human primates, such as *Macaca sp.* monkeys (Moyes et al. 2016). This is a particular problem in Malaysian Borneo, where a risk map was developed to visualise land use and assess malaria risk distributions (Sato et al. 2019). Such mapping approaches can help determine the risk factor for vector‐spread protozoa under LUC, enabling people to predict and mitigate outbreaks.

### Anthropogenic land‐use changes

Incursions into wild habitats expose humans to new pathogens if they come into contact with wild animals or hunt, butcher, and consume wild meat (Cantlay et al. 2017). Agricultural land can be used for food‐animal production, which brings domestic animals physically closer to other individuals and into frequent contact with humans. If biosecurity methods are not applied, this can impact the rate and pattern of zoonotic disease spread (Jones et al. 2013). Some forms of land use can alter entire ecosystems. Responding to these changes demands fast adaptations of wild animals' foraging strategies and use of space (Jung & Kalko 2010), which often bring wildlife into closer and more frequent contact with humans, thus increasing the chance of pathogen transmission and changing patterns of zoonotic EID spread. Resource provisioning in human‐dominated habitats can also affect infection outcomes in wildlife, increasing levels of infection by helminths and viruses (Becker et al. 2015).

The most commonly studied LUC in the mammalian dataset was urbanisation (61 studies; 45%), followed by agricultural intensification (31; 23%), deforestation (20; 15%), and habitat fragmentation (17, 13%; Fig. 2c). Of the remaining studies, 14 discussed LUC in general and three covered impacts of other LUCs, including woodland expansion (Millins et al. 2017) and watershed development (Walker et al. 2008).

#### Urbanisation

More than half of the human population inhabits urban settlements, and cities are projected to increase in both size and number as the human population expands (United Nations 2016). This rapid LUC will lead to new challenges for global health and epidemiology of zoonotic EIDs, given evidence of increased transmission in urban‐adapted hosts, such as rodents. Urbanisation can provide favourable eco‐epidemiological conditions for rodent‐borne *Leptospira* spp. that is becoming an emerging risk and a serious threat in urbanised areas in both developing and developed countries (Kurucz et al. 2018, Blasdell et al. 2019). Rats and urbanisation have also been associated with increased spread of bartonella in North America (Peterson et al. 2017, Rothenburger et al. 2018) and leishmaniasis in Borneo and Brazil (Shapiro et al. 2013). Moreover, helminths from foxes are spreading partly as a result of urbanisation in Europe (Pavlovic et al. 1997, Otero‐Abad et al. 2017). It remains unclear whether this occurs globally, but it has been reported in other carnivores (Otranto & Deplazes 2019). The 2006 influenza A H1N1 (swine flu) urban pandemic shows just how fast a zoonotic disease can spread and become uncontrollable in the absence of containment provisions (Fasina et al. 2007). This is an example of how outbreaks could be a greater threat in the future, as new megacities could become incubators of zoonotic diseases that will allow them to spread faster and become a worldwide threat (Neiderud 2015). The association between carnivores and zoonotic helminths also increases in urban areas (Field 2009). Bats can form large roosts even in dense urban centres (Hayman et al. 2012), yet only three of the studies reviewed addressed impacts of urbanisation on bat pathogens (Field 2009, Shapiro et al. 2013, Pernet et al. 2014). As bats are important reservoirs for zoonotic diseases (Allocati et al. 2016), it is essential to understand how urbanisation may affect the risk of disease spread.

#### Agricultural intensification

The most important infectious human diseases have come into existence since the advent of agriculture, and in particular since the domestication of animals (Carroll et al. 2010). As the human population continues to rise, there will be an increasing dependency on agricultural systems to provide food and other resources. Rapid growth in meat consumption increases the chance of exposing consumers to food‐borne pathogens, particularly from chickens and pigs (CIWF 2013, Gilbert et al. 2015). Industrial food animal production systems increase animal and public health risks as they create diverse wildlife–livestock–human interfaces (Jones et al. 2013, Hassell et al. 2017), increasing the risk of zoonotic emergence as agriculture intensifies. These industrial systems involve keeping a large number of animals confined to a small space in close physical contact, where pathogens can easily be transmitted. Risks are particularly high for large‐scale livestock farm workers and neighbouring residents, who can be exposed to harmful bacteria and viruses (Smit & Heederik 2017).

In developed countries, tuberculosis outbreaks are mitigated by strict animal control, elimination programmes and milk pasteurisation, as well as access to veterinary services, which reduces the chances of transmission to humans (Cosivi et al. 1998). However, in Indian dairy farms, it has been found that selling or abandoning infected animals, lack of education about bovine tuberculosis, and only consulting veterinarians as a last resort worsens the problem (Chauhan et al. 2019). As industrial food‐animal production becomes increasingly common in developing countries, agricultural intensification is likely to increase the risk of zoonotic disease emergence and spread. Differences in farming practices between countries as a result of culture or income can lead to differences in outbreak patterns, posing challenges for research (Gilbert et al. 2015).

#### Deforestation and habitat fragmentation

Deforestation is considered the most immediate contributor to the likelihood of zoonotic disease emergence and spread, as natural forest ecosystems are disrupted through habitat destruction, habitat fragmentation, and conversion into anthropogenic environments (Sehgal 2010). An estimated 1.6 billion people rely on forests for survival (Anonymous 2018), and risks can occur when humans come into contact with wildlife and are exposed to new pathogens. In addition, the formation of forest edges affects the ecology of zoonotic diseases by providing the opportunity for local epidemic expansions (Sharma & Kondrashin 1991).

The purpose of deforestation is often logging activities. The mechanisms of pathogen transmission are complex and differ with logging method. The low contact rate between humans and wildlife during clear‐cut logging reduces the chance of zoonotic emergence compared with selective extraction, the favoured method used in Central African logging (Fa et al. 1995). However, regardless of the method, the removal of trees still drastically reshapes the environment, transforming whole ecosystems, and consequently affecting disease emergence and transmission (Taylor 1997). Conversion of forests to agricultural land results in decreased diversity of zoonotic microparasites and rodent‐borne pathogens in South‐East Asia; however, the consequent increase in synanthropic rodents favours pathogen spread (Morand et al. 2019). In South America, where deforestation rates are high, there are instances of zoonotic EIDs including microsporidia (Pereira et al. 2009), *Bartonella*, and *Leptospira* (Cortez et al. 2018). However, it is not clear whether microsporidia spores found in wild mammal faeces are always the result of an infection, rather than simply passing through the gastrointestinal tract (Pereira et al. 2009), and the methodology used for *Bartonella* and *Leptospira* identification has limitations (Cortez et al. 2018). Therefore, the prevalence of these zoonoses in areas with deforestation is not yet well understood.

Deforestation has been associated with the increased emergence of pathogens in bats around the world, due to the creation of patches of habitat that isolate or divide populations, alter behaviour, reduce biodiversity, and compromise ecosystem functions (Willig et al. 2019). Viruses of notable concern include henipaviruses in Africa (Pernet et al. 2014), Hendra virus in Australia (Wild 2009), and Nipah virus in Malaysia (Field 2009). Likewise, non‐human primates show an increase in zoonotic parasites with fragmentation of forests (Gillespie 2006); parasites include plasmodium (Moyes et al. 2016, Sato et al. 2019) and a range of helminth species (Klaus et al. 2017). In Sri Lanka, habitat fragmentation from deforestation has led to wild animals roaming in nearby neighbourhoods, increasing the exposure of residents to ticks and the risk of tick‐borne infections (Liyanaarachchi et al. 2015).

There is further uncertainty about how vector‐borne disease emergence will change with forest clearance. Some studies show that the loss of forests may eliminate local vector species (Molyneux 2003), whereas woodland expansion was found to increase suitable habitat for hosts and the tick vectors of Lyme disease, and may therefore increase risk of emergence (Millins et al. 2017). Other researchers warn of a higher risk of infection for people residing near fragmented forests because vector species find new breeding sites by reshaping ecosystem boundaries, which are often points of contact between humans and pathogens (Gottwalt 2015). For example, Brazilian spotted fever, caused by the bacterium *Rickettsia rickettsia* and spread by the tick *Amblyomma cajennense*, is associated with habitat fragmentation and lower abundance and richness of wild vertebrates (Scinachi et al. 2017).

#### Multiple land‐use changes

The spread of some zoonoses has been associated with the impacts of multiple LUCs. The spread of *Bartonella,* the bacterium causing bartonellosis, increases with deforestation (Cortez et al. 2018, Neves et al. 2018) and urbanisation (Peterson et al. 2017, Rothenburger et al. 2018), and outbreaks associated with these LUCs have been identified in South America, North America, and Asia. Similarly, the prevalence of the helminth *Echinococcus multilocularis* in hosts increases with both deforestation (Giraudoux et al. 2003) and urbanisation (Fischer et al. 2005).

### Comparison with previous reviews

A previous review by Gottdenker et al. (2014) covering changes in spread of EIDs under anthropogenic LUC up to the year 2012 identified, similar to our review, agricultural development, urbanisation, and deforestation or habitat fragmentation as key LUCs. Although both reviews identified leishmaniasis and Lyme disease as commonly studied pathogens, our focus on mammals meant that *Echinococcus* and leptospirosis studies were more common than malaria and Chagas disease studies. Our review further considers the region where each study took place, and found differences in study frequency of each pathogen or land‐use type between continents. We also consider the host taxon for each paper and find that this changes between pathogen type, LUC type, and region, whereas Gottdenker et al. (2014) only identify whether the pathogen studied was multi‐host or single host. Other previous reviews were focused on specific land‐use types. For example, Hassell et al. (2017) reviewed the link between urbanisation and disease emergence dynamics at the wildlife–livestock–human interface. They showed that most urban disease transmission studies were focused on a single species and pathogen or on a small number of species and pathogens, which alone may not be suitable for understanding epidemiology. Finally, Han et al. (2016) present a more general review of zoonotic disease in mammals, mapping global patterns of disease risk, identifying rodents, carnivores, and ungulates (especially livestock) as having highest zoonotic potential, and associating carnivores with zoonotic bacteria pathogens, rodents with helminths, and ungulates with protozoa. However, they do not review the link between zoonotic disease spread and anthropogenic LUC, though they do mention the importance of understanding extrinsic pressures that influence disease outbreaks in humans.

### Future research needs

Predicting how zoonotic diseases emerge and spread in response to anthropogenic LUC requires a comprehensive understanding of how these changes will influence both the hosts and the pathogens. For each of the identified LUCs, the recognition of patterns and consistency of emergences require reliable surveillance and an understanding of transmission, but our results show that this information is not yet available for all hosts and pathogens. Multiple pathogens in rodent reservoirs identified by the Centre for Disease Control and Prevention have not been the subject of research, such as Lassa fever (CDC 2017). Similarly, some key zoonoses hosted by bats have been understudied within the context of impacts of LUC, including coronaviruses (only two studies). Rarer diseases in livestock are also missing epidemiology and surveillance data, for example anthrax, and emerging diseases such as Q fever require more attention.

Our results show that it is not fully understood how parasitic nematodes in carnivores are spread globally, particularly in urban environments. In contrast, primate studies predominantly covered infections by nematodes (Arizono et al. 2012, Klaus et al. 2017, Rondon et al. 2017), and less research attention has been given to pathogens such as viruses. In fact, the epidemiology of many zoonotic viruses is yet to be considered in relation to LUC. In addition, studies into both reforestation and habitat fragmentation identified an increased risk of Lyme disease (Millins et al. 2017), suggesting that further investigation is needed into the best way to mitigate outbreaks following deforestation.

Understanding how zoonotic diseases emerge and spread in response to LUC requires adequate identification of the incidence of infection. As noted in the papers collected, appropriate tests to identify infection are not always available. For example, the frequently used test for microsporidia infection, the presence of eggs in faecal matter, is not necessarily indicative of infection (Pereira et al. 2009).

Our review highlights new emerging approaches to the study of effects of LUC on the spread of mammalian zoonotic diseases, such as risk maps developed for malaria (Sato et al. 2019). Similarly, modelling approaches are promising tools for identifying general trends and predicting future consequences, yet we found that they are underutilised.

There is an urgent need for empirical studies that link host ecology and responses to LUC with epidemiology and patterns of disease spread. Although the majority of mammalian studies reviewed were empirical, more than 80% of studies looking at impacts of agriculture, a major driver of LUC, were reviews. Moreover, there is a need for data synthesis studies, such as global‐scale meta‐analyses or applications of data science methods, to identify whether the different LUCs have consistent impacts, in terms of either the pathogen groups or host taxa studied.

## Conclusions

This systematic review identified key hosts, pathogens, and LUC categories covered in the literature on the effect of anthropogenic LUC on the spread of mammalian emerging zoonotic diseases, and their geographic distribution and interactions. The studies we reviewed suggest that the direct and knock‐on effects of anthropogenic LUC are likely to increase the spread of EIDs. Yet, several gaps in the literature limit our understanding of how zoonotic disease spread and host–pathogen interactions may change in response to LUC. Gaining a more comprehensive understanding of how anthropogenic LUC affects the spread of emerging zoonotic diseases is essential for predicting and mitigating future emergences through fine‐tuning surveillance and control measures towards particular locations and reservoirs. The link between anthropogenic impacts on the natural environment and the recent COVID‐19 pandemic (Zhang & Holmes 2020) highlights the urgent need to increase understanding of how anthropogenic LUC affects the risk of spillover to humans and spread of zoonotic diseases.

## Supporting information


**Appendix S1.** List of papers obtained from the systematic review search string.

**Appendix S2.** Data extracted from the papers for analysis.

## References

[mam12201-bib-0001] Abera E , Assefa A , Belete S , Mekonen N (2015) Review on rabies, with emphasis on disease control and eradication measures. International Journal of Basic and Applied Virology 4: 60–70.

[mam12201-bib-0002] Abuzaid AA , Abdoon AM , Aldahan MA , Alzahrani AG , Alhakeem RF , Asiri AM , Alzahrani MH , Memish ZA (2017) Cutaneous leishmaniasis in Saudi Arabia: a comprehensive overview. Vector‐Borne and Zoonotic Diseases 17: 673–684. 28806141 10.1089/vbz.2017.2119 PMC5649416

[mam12201-bib-0003] Allen T , Murray KA , Zambrana‐Torrelio C , Morse SS , Rondinini C , Di Marco M , Breit N , Olival KJ , Daszak P (2017) Global hotspots and correlates of emerging zoonotic diseases. Nature Communications 8: 1124. 10.1038/s41467-017-00923-8 PMC5654761 29066781

[mam12201-bib-0004] Allocati N , Petrucci AG , Di Giovanni P , Masulli M , Di Ilio C , De Laurenzi V (2016) Bat‐man disease transmission: zoonotic pathogens from wildlife reservoirs to human populations. Cell Death Discovery 2: 16048. 27551536 10.1038/cddiscovery.2016.48 PMC4979447

[mam12201-bib-0005] Anders JL , Nakao M , Uchida K , Ayer CG , Asakawa M , Koizumi I (2019) Comparison of the intestinal helminth community of the large Japanese field mouse (*Apodemus speciosus*) between urban, rural, and natural sites in Hokkaido, Japan. Parasitology International 70: 51–57. 30716461 10.1016/j.parint.2019.02.001

[mam12201-bib-0006] Anonymous (2018) At the human‐forest interface. Editorial. Nature Communications 9: 1153. 10.1038/s41467-018-03586-1 PMC5862979 29563499

[mam12201-bib-0007] Arizono N , Yamada M , Tegoshi T , Onishi K (2012) Molecular identification of *Oesophagostomum* and *Trichuris* eggs isolated from wild Japanese macaques. Korean Journal of Parasitology 50: 253–257. 22949756 10.3347/kjp.2012.50.3.253 PMC3428574

[mam12201-bib-0008] Baily JL , Méric G , Bayliss S , Foster G , Moss SE , Watson E et al. (2015) Evidence of land‐sea transfer of the zoonotic pathogen *Campylobacter* to a wildlife marine sentinel species. Molecular Ecology 24: 208–221. 25401947 10.1111/mec.13001

[mam12201-bib-0009] Bayry J (2013) Emerging viral diseases of livestock in the developing world. Indian Journal of Virology 24: 291–294. 24426290 10.1007/s13337-013-0164-x PMC3832702

[mam12201-bib-0010] Becker DJ , Streicker DG , Altizer S (2015) Linking anthropogenic resources to wildlife–pathogen dynamics: a review and meta‐analysis. Ecology Letters 18: 483–495. 25808224 10.1111/ele.12428 PMC4403965

[mam12201-bib-0011] Blasdell KR , Morand S , Perera D , Firth C (2019) Association of rodent‐borne *Leptospira* spp. with urban environments in Malaysian Borneo. PLoS Neglected Tropical Diseases 13: e0007141. 30811387 10.1371/journal.pntd.0007141 PMC6411199

[mam12201-bib-0012] Bordes F , Morand S , Pilosof S , Claude J , Krasnov BR , Cosson J et al. (2015) Habitat fragmentation alters the properties of a host‐parasite network: rodents and their helminths in South‐East Asia. Journal of Animal Ecology 84: 1253–1263. 25777342 10.1111/1365-2656.12368

[mam12201-bib-0013] Brook CE , Dobson AP (2015) Bats as ‘special’ reservoirs for emerging zoonotic pathogens. Trends in Microbiology 23: 172–180. 25572882 10.1016/j.tim.2014.12.004 PMC7126622

[mam12201-bib-0014] Cantlay JC , Ingram DJ , Meredith AL (2017) A review of zoonotic infection risks associated with the wild meat trade in Malaysia. EcoHealth 14: 361–388. 28332127 10.1007/s10393-017-1229-x PMC5486459

[mam12201-bib-0015] Carroll SA , Bird BH , Rollin PE , Nichol ST (2010) Ancient common ancestry of Crimean‐Congo hemorrhagic fever virus. Molecular Phylogenetics and Evolution 55: 1103–1110. 20074652 10.1016/j.ympev.2010.01.006

[mam12201-bib-0016] Carver S , Spafford H , Storey A , Weinstein P (2009) Dryland salinity and the ecology of Ross River Virus: the ecological underpinnings of the potential for transmission. Vector‐Borne and Zoonotic Diseases 9: 611–622. 19326966 10.1089/vbz.2008.0124

[mam12201-bib-0017] Cascio A , Bosilkovski M , Rodriguez‐Morales AJ , Pappas G (2011) The socio‐ecology of zoonotic infections. Clinical Microbiology and Infection 17: 336–342. 21175957 10.1111/j.1469-0691.2010.03451.x

[mam12201-bib-0018] CDC (2017) Diseases directly transmitted by rodents. https://www.cdc.gov/rodents/diseases/direct.html [accessed 11 December 2019].

[mam12201-bib-0019] CDC (2019) Lyme and other tickborne diseases increasing. https://www.cdc.gov/media/dpk/diseases‐and‐conditions/lyme‐disease/index.html [accessed 14 December 2019].

[mam12201-bib-0020] Chauhan AS , George MS , Lindahl J , Grace D , Kakkar M (2019) Community, system and policy level drivers of bovine tuberculosis in smallholder periurban dairy farms in India: a qualitative enquiry. BMC Public Health 19: 301. 30866894 10.1186/s12889-019-6634-3 PMC6415345

[mam12201-bib-0021] CIWF (2013) Zoonotic Diseases Human Health and Farm Animal Welfare Report. https://www.ciwf.org.uk/media/3756123/Zoonotic‐diseases‐human‐health‐and‐farm‐animal‐welfare‐16‐page‐report.pdf [accessed January 2019].

[mam12201-bib-0022] Clark NJ , Soares Magalhães RJ (2018) Airborne geographical dispersal of Q fever from livestock holdings to human communities: a systematic review and critical appraisal of evidence. BMC Infectious Diseases 18: 218. 29764368 10.1186/s12879-018-3135-4 PMC5952368

[mam12201-bib-0023] Cortez V , Canal E , Dupont‐Turkowsky JC , Quevedo T , Albujar C , Chang T‐C et al. (2018) Identification of *Leptospira* and *Bartonella* among rodents collected across a habitat disturbance gradient along the Inter‐Oceanic Highway in the southern Amazon Basin of Peru. PLoS ONE 13: e0205068. 30300359 10.1371/journal.pone.0205068 PMC6177132

[mam12201-bib-0024] Cosivi O , Grange JM , Daborn CJ , Raviglione MC , Fujikura T , Cousins D et al. (1998) Zoonotic tuberculosis due to *Mycobacterium bovis* in developing countries. Emerging Infectious Diseases 4: 59–70. 9452399 10.3201/eid0401.980108 PMC2627667

[mam12201-bib-0025] DiEuliis D , Johnson KR , Morse SS , Schindel DE (2016) Opinion: specimen collections should have a much bigger role in infectious disease research and response. Proceedings of the National Academy of Sciences USA 113: 4–7. 10.1073/pnas.1522680112 PMC4711881 26733667

[mam12201-bib-0026] Dobson AP , Carper ER (1996) Infectious diseases and human population history. BioScience 46: 115–126.

[mam12201-bib-0027] Domingo E , Holland JJ (1997) RNA virus mutations and fitness for survival. Annual Review of Microbiology 51: 151–178. 10.1146/annurev.micro.51.1.151 9343347

[mam12201-bib-0028] Donalisio MR , Paiz LM , da Silva VG , Richini‐Pereira VB , von Zuben APB , Castagna CL , Motoie G , Hiramoto RM , Tolezano JE (2017) Visceral leishmaniasis in an environmentally protected area in southeastern Brazil: epidemiological and laboratory cross‐sectional investigation of phlebotomine fauna, wild hosts and canine cases. PLoS Neglected Tropical Diseases 11: e0005666. 28704391 10.1371/journal.pntd.0005666 PMC5509102

[mam12201-bib-0029] Ducrotoy MJ , Bertu WJ , Ocholi RA , Gusi AM , Bryssinckx W , Welburn S , Moriyón I (2014) Brucellosis as an emerging threat in developing economies: lessons from Nigeria. PLoS Neglected Tropical Diseases 8: e3008. 25058178 10.1371/journal.pntd.0003008 PMC4109902

[mam12201-bib-0030] Duffy S , Shackelton LA , Holmes EC (2008) Rates of evolutionary change in viruses: patterns and determinants. Nature Reviews Genetics 9: 267–276. 10.1038/nrg2323 18319742

[mam12201-bib-0031] Duplantier JM , Duchemin JB , Chanteau S , Carniel E (2005) From the recent lessons of the Malagasy foci towards a global understanding of the factors involved in plague re‐emergence. Veterinary Research 36: 437–453. 15845233 10.1051/vetres:2005007

[mam12201-bib-0032] Fa JE , Juste J , Del Val JP , Castroviejo J (1995) Impact of market hunting on mammal species in Equatorial Guinea. Conservation Biology 9: 1107–1115. 34261280 10.1046/j.1523-1739.1995.951107.x

[mam12201-bib-0033] Fasina FO , Bisschop SP , Webster RG (2007) Avian influenza H5N1 in Africa: an epidemiological twist. Lancet Infectious Diseases 7: 696–697. 17961853 10.1016/S1473-3099(07)70244-X

[mam12201-bib-0034] Field HE (2009) Bats and emerging zoonoses: henipaviruses and SARS. Zoonoses and Public Health 56: 278–284. 19497090 10.1111/j.1863-2378.2008.01218.x

[mam12201-bib-0035] Fischer C , Reperant LA , Weber JM , Hegglin D , Deplazes P (2005) *Echinococcus multilocularis* infections of rural, residential and urban foxes (*Vulpes vulpes*) in the canton of Geneva, Switzerland. Parasite‐Journal De La Societe Francaise De Parasitologie 12: 339–346. 16402566 10.1051/parasite/2005124339

[mam12201-bib-0036] Gilbert M , Conchedda G , Van Boeckel TP , Cinardi G , Linard C , Nicolas G et al. (2015) Income disparities and the global distribution of intensively farmed chicken and pigs. PLoS ONE 10: e0133381. 26230336 10.1371/journal.pone.0133381 PMC4521704

[mam12201-bib-0037] Gillespie TR (2006) Noninvasive assessment of gastrointestinal parasite infections in free‐ranging primates. International Journal of Primatology 27: 1129–1143.

[mam12201-bib-0038] Giraudoux P , Ps C , Delattre P , Bao G , Bartholomot B , Harraga S et al. (2003) Interactions between landscape changes and host communities can regulate *Echinococcus multilocularis* transmission. Parasitology 127: S119–S129. 15027609

[mam12201-bib-0039] Gottdenker NL , Streicker DG , Faust CL , Carroll CR (2014) Anthropogenic land‐use change and infectious diseases: a review of the evidence. EcoHealth 11: 619–632. 24854248 10.1007/s10393-014-0941-z

[mam12201-bib-0040] Gottwalt A (2015) Impacts of deforestation on vector‐borne disease incidence. Global Journal of Health Science 3: 16–19.

[mam12201-bib-0041] Han BA , Kramer AM , Drake JM (2016) Global patterns of zoonotic disease in mammals. Trends in Parasitology 32: 565–577. 27316904 10.1016/j.pt.2016.04.007 PMC4921293

[mam12201-bib-0042] Hancke D , Suarez OV (2018) Structure of parasite communities in urban environments: the case of helminths in synanthropic rodents. Folia Parasitologica 65. 10.14411/fp.2018.009 30183669

[mam12201-bib-0043] Hassell JM , Begon M , Ward MJ , Fevre EM (2017) Urbanization and disease emergence: dynamics at the wildlife‐livestock‐human interface. Trends in Ecology and Evolution 32: 55–67. 28029378 10.1016/j.tree.2016.09.012 PMC5214842

[mam12201-bib-0044] Hayman DTS , Bowen RA , Cryan PM , McCracken GF , O'Shea TJ , Peel AJ , Gilbert A , Webb CT , Wood JLN (2012) Ecology of zoonotic infectious diseases in bats: current knowledge and future directions. Zoonoses and Public Health 60: 2–21. 22958281 10.1111/zph.12000 PMC3600532

[mam12201-bib-0045] HSE (2019) Zoonoses. https://www.hse.gov.uk/agriculture/topics/zoonoses.htm [accessed 11 December 2019].

[mam12201-bib-0046] Johnson CK , Hitchens PL , Evans TS , Goldstein T , Thomas K , Clements A et al. (2015) Spillover and pandemic properties of zoonotic viruses with high host plasticity. Nature Science Reports 5: 14830. 10.1038/srep14830 PMC4595845 26445169

[mam12201-bib-0047] Jones KE , Patel NG , Levy MA , Storeygard A , Balk D , Gittleman JL , Daszak P (2008) Global trends in emerging infectious diseases. Nature 451: 990–993. 18288193 10.1038/nature06536 PMC5960580

[mam12201-bib-0048] Jones BA , Grace D , Kock R , Alonso S , Rushton J , Said MY et al. (2013) Zoonosis emergence linked to agricultural intensification and environmental change. Proceedings of the National Academy of Sciences USA 110: 8399–8404. 10.1073/pnas.1208059110 PMC3666729 23671097

[mam12201-bib-0049] Jung K , Kalko EKV (2010) Where forest meets urbanization: foraging plasticity of aerial insectivorous bats in an anthropogenically altered environment. Journal of Mammalogy 91: 144–153.

[mam12201-bib-0050] Kessler MK , Becker DJ , Peel AJ , Justice NV , Lunn T , Crowley DE et al. (2018) Changing resource landscapes and spillover of henipaviruses. Annals of the New York Academy of Sciences 1429: 78–99. 30138535 10.1111/nyas.13910 PMC6778453

[mam12201-bib-0051] Klaus A , Zimmermann E , Roper KM , Radespiel U , Nathan S , Goossens B , Strube C (2017) Co‐infection patterns of intestinal parasites in arboreal primates (proboscis monkeys, *Nasalis larvatus*) in Borneo. International Journal for Parasitology‐Parasites and Wildlife 6: 320–329. 29988805 10.1016/j.ijppaw.2017.09.005 PMC6031963

[mam12201-bib-0052] Ko AI , Goarant C , Picardeau M (2009) *Leptospira*: the dawn of the molecular genetics era for an emerging zoonotic pathogen. Nature Reviews Microbiology 7: 736–747. 19756012 10.1038/nrmicro2208 PMC3384523

[mam12201-bib-0053] Kurucz K , Madai M , Bali D , Hederics D , Horváth G , Kemenesi G , Jakab F (2018) Parallel survey of two widespread renal syndrome‐causing zoonoses: *Leptospira* spp. and *Hantavirus* in urban environment, Hungary. Vector‐Borne and Zoonotic Diseases 18: 200–205. 29437551 10.1089/vbz.2017.2204

[mam12201-bib-0054] Lallo MA , Pereira A , Araujo R , Favorito SE , Bertolla P , Bondan EF (2009) Occurrence of *Giardia*, *Cryptosporidium* and microsporidia in wild animals from a deforestation area in the state of Sao Paulo, Brazil. Ciencia Rural 39: 1465–1470.

[mam12201-bib-0055] Lederberg J , Shope RE , Oakes SC (1992) Emerging Infections: Microbial Threats to Health in the United States. Institute of Medicine Committee on Emerging Microbial Threats to Health, National Academies Press, Washington DC, USA. 25121245

[mam12201-bib-0056] Lendak D , Preveden T , Kovacevic N , Tomic S , Ruzic M , Fabri M (2017) Novel infectious diseases in Europe. Medical Review 70: 385–390.

[mam12201-bib-0057] Levett P (2001) Leptospirosis. Clinical Microbiology Reviews 14: 296–326. 11292640 10.1128/CMR.14.2.296-326.2001 PMC88975

[mam12201-bib-0058] Liyanaarachchi DR , Rajakaruna RS , Dikkumbura AW , Rajapakse R (2015) Ticks infesting wild and domestic animals and humans of Sri Lanka with new host records. Acta Tropica 142: 64–70. 25445744 10.1016/j.actatropica.2014.11.001

[mam12201-bib-0059] Luis AD , Hayman DTS , O'Shea TJ , Cryan PM , Gilbert AT , Pulliam JRC et al. (2013) A comparison of bats and rodents as reservoirs of zoonotic viruses: are bats special? Proceedings of the Royal Society B 280: 20122753. 23378666 10.1098/rspb.2012.2753 PMC3574368

[mam12201-bib-0060] Luis AD , O'Shea TJ , Hayman DTS , Wood JLN , Cunningham AA , Gilbert AT , Mills JN , Webb CT (2015) Network analysis of host–virus communities in bats and rodents reveals determinants of cross‐species transmission. Ecology Letters 18: 1153–1162. 26299267 10.1111/ele.12491 PMC5014217

[mam12201-bib-0061] Maaz D , Krücken J , Blümke J , Richter D , McKay‐Demeler J , Matuschka F‐R , Hartmann S , von Samson‐Himmelstjerna G (2018) Factors associated with diversity, quantity and zoonotic potential of ectoparasites on urban mice and voles. PLoS ONE 13: e0199385. 29940047 10.1371/journal.pone.0199385 PMC6016914

[mam12201-bib-0062] Mackenstedt U , Jenkins D , Romig T (2015) The role of wildlife in the transmission of parasitic zoonoses in peri‐urban and urban areas. International Journal for Parasitology: Parasites and Wildlife 4: 71–79. 25830108 10.1016/j.ijppaw.2015.01.006 PMC4356871

[mam12201-bib-0063] Manangan JS , Schweitzer SH , Nibbelink N , Yabsley MJ , Gibbs SEJ , Wimberly MC (2007) Habitat factors influencing distributions of *Anaplasma phagocytophilum* and *Ehrlichia chaffeensis* in the Mississippi alluvial valley. Vector‐Borne and Zoonotic Diseases 7: 563–573. 18047394 10.1089/vbz.2007.0116

[mam12201-bib-0064] Marston CG , Danson FM , Armitage RP , Giraudoux P , Pleydell DRJ , Wang Q , Qui J , Craig PS (2014a) A random forest approach for predicting the presence of *Echinococcus multilocularis* intermediate host *Ochotona* spp. presence in relation to landscape characteristics in western China. Applied Geography 55: 176–183. 25386042 10.1016/j.apgeog.2014.09.001 PMC4223806

[mam12201-bib-0065] Marston HD , Folkers GK , Morens DM , Fauci AS (2014b) Emerging viral diseases: confronting threats with new technologies. Science Translational Medicine 6: 253ps10. 10.1126/scitranslmed.3009872 25210060

[mam12201-bib-0066] Marzetti S , Carranza C , Roncallo M , Escobar GI , Lucero NE (2013) Recent trends in human *Brucella canis* infection. Comparative Immunology, Microbiology and Infectious Diseases 36: 55–61. 23040615 10.1016/j.cimid.2012.09.002

[mam12201-bib-0067] Mehndiratta P , Bhalla P (2014) Use of antibiotics in animal agriculture and emergence of methicillin‐resistant *Staphylococcus aureus* (MRSA) clones: need to assess the impact on public health. Indian Journal of Medical Research 140: 339–344. 25366200 PMC4248379

[mam12201-bib-0068] Millán J , Proboste T , Fernández de Mera IG , Chirife AD , de la Fuente J , Altet L (2016) Molecular detection of vector‐borne pathogens in wild and domestic carnivores and their ticks at the human–wildlife interface. Ticks and Tick‐borne Diseases 7: 284–290. 26643497 10.1016/j.ttbdis.2015.11.003

[mam12201-bib-0069] Millins C , Gilbert L , Medlock J , Hansford K , Thompson DBA , Biek R (2017) Effects of conservation management of landscapes and vertebrate communities on Lyme borreliosis risk in the United Kingdom. Philosophical Transactions of the Royal Society B: Biological Sciences 372: 20160123. 10.1098/rstb.2016.0123 PMC5413871 28438912

[mam12201-bib-0070] Millins C , Dickinson ER , Isakovic P , Gilbert L , Wojciechowska A , Paterson V et al. (2018) Landscape structure affects the prevalence and distribution of a tick‐borne zoonotic pathogen. Parasites & Vectors 11: 621. 30514350 10.1186/s13071-018-3200-2 PMC6278045

[mam12201-bib-0071] Moher D , Liberati A , Tetzlaff J , Altman DG (2009) Preferred reporting items for systematic reviews and meta‐analyses: the PRISMA statement. BMJ 339: b2535. 19622551 10.1136/bmj.b2535 PMC2714657

[mam12201-bib-0072] Molyneux DH (2003) Common themes in changing vector‐borne disease scenarios. Transactions of the Royal Society of Tropical Medicine and Hygiene 97: 129–132. 14584362 10.1016/s0035-9203(03)90097-6

[mam12201-bib-0073] Morand S , Blasdell K , Bordes FE , Buchy P , Carcy B , Chaisiri K et al. (2019) Changing landscapes of Southeast Asia and rodent‐borne diseases: decreased diversity but increased transmission risks. Ecological Applications 29: e01886. 30986339 10.1002/eap.1886

[mam12201-bib-0074] Morse SS , Mazet JAK , Woolhouse M , Parrish CR , Carroll D , Karesh WB , Zambrana‐Torrelio C , Lipkin I , Daszak P (2012) Prediction and prevention of the next pandemic zoonosis. The Lancet 380: 1956–1965. 10.1016/S0140-6736(12)61684-5 PMC3712877 23200504

[mam12201-bib-0075] Moyes CL , Shearer FM , Huang Z , Wiebe A , Gibson HS , Nijman V et al. (2016) Predicting the geographical distributions of the macaque hosts and mosquito vectors of *Plasmodium knowlesi* malaria in forested and non‐forested areas. Parasites & Vectors 9: 242. 27125995 10.1186/s13071-016-1527-0 PMC4850754

[mam12201-bib-0076] Neiderud C‐J (2015) How urbanization affects the epidemiology of emerging infectious diseases. Infection Ecology and Epidemiology 5: 27060. 26112265 10.3402/iee.v5.27060 PMC4481042

[mam12201-bib-0077] Neves ES , Mendenhall IH , Borthwick SA , Su YCF , Smith GJD (2018) Detection and genetic characterization of diverse *Bartonella* genotypes in the small mammals of Singapore. Zoonoses and Public Health 65: E207–E215. 29235263 10.1111/zph.12430

[mam12201-bib-0078] Nunes H , Rocha FL , Cordeiro‐Estrela P (2017) Bats in urban areas of Brazil: roosts, food resources and parasites in disturbed environments. Urban Ecosystems 20: 953–969. 32214783 10.1007/s11252-016-0632-3 PMC7089172

[mam12201-bib-0079] Okello AL , Burniston S , Conlan JV , Inthavong P , Khamlome B , Welburn SC , Gilbert J , Allen J , Blacksell SD (2015) Prevalence of endemic pig‐associated zoonoses in Southeast Asia: a review of findings from the Lao People's Democratic Republic. American Journal of Tropical Medicine and Hygiene 92: 1059–1066. 25802431 10.4269/ajtmh.14-0551 PMC4426289

[mam12201-bib-0080] de Oliveira AC , Figueiredo FB , Silva VL , Santos FN , de Souza MB , Madeira MD , Abrantes TR , Perisse ARS (2015) Canine visceral leishmaniasis case investigation in the Jacare region on Niteroi, Rio de Janeiro, Brazil. Revista Do Instituto De Medicina Tropical De Sao Paulo 57: 325–332. 26422157 10.1590/S0036-46652015000400009 PMC4616918

[mam12201-bib-0081] Otero‐Abad B , Rüegg SR , Hegglin D , Deplazes P , Torgerson PR (2017) Mathematical modelling of *Echinococcus multilocularis* abundance in foxes in Zurich, Switzerland. Parasites & Vectors 10: 21. 28077161 10.1186/s13071-016-1951-1 PMC5225524

[mam12201-bib-0082] Otranto D , Deplazes P (2019) Zoonotic nematodes of wild carnivores. International Journal for Parasitology‐Parasites and Wildlife 9: 370–383. 31338295 10.1016/j.ijppaw.2018.12.011 PMC6626844

[mam12201-bib-0083] Parker S , Nuara A , Buller RML , Schultz DA (2007) Human monkeypox: an emerging zoonotic disease. Future Microbiology 2: 17–34. 17661673 10.2217/17460913.2.1.17

[mam12201-bib-0084] Pavlovic I , Kulisic Z , Milutinovic M (1997) The role of foxes (*Vulpes vulpes* L.) in the epizootiology and epidemiology of nematode parasitic zoonoses. Acta Veterinaria‐Beograd 47: 177–182.

[mam12201-bib-0085] Pereira A , Araujo RS , Favorito SE , Bertolla PB , Lallo MA (2009) Occurrence of microsporidia in small wildlife mammals in the state of Sao Paulo, Brazil. Arquivo Brasileiro De Medicina Veterinaria E Zootecnia 61: 1474–1477.

[mam12201-bib-0086] Pernet O , Schneider BS , Beaty SM , LeBreton M , Yun TE , Park A et al. (2014) Evidence for *Henipavirus* spillover into human populations in Africa. Nature Communications 5: 5342. 10.1038/ncomms6342 PMC4237230 25405640

[mam12201-bib-0087] Peterson AC , Ghersi BM , Alda F , Firth C , Frye MJ , Bai Y et al. (2017) Rodent‐borne *Bartonella* infection varies according to host species within and among cities. EcoHealth 14: 771–782. 29164472 10.1007/s10393-017-1291-4

[mam12201-bib-0088] Plowright RK , Parrish CR , McCallum H , Hudson PJ , Ko AI , Graham AL et al. (2017) Pathways to zoonotic spillover. Nature Reviews Microbiology 15: 502–510. 28555073 10.1038/nrmicro.2017.45 PMC5791534

[mam12201-bib-0089] Pulliam JRC , Epstein JH , Dushoff J , Rahman SA , Bunning M , Jamaluddin AA et al. (2012) Agricultural intensification, priming for persistence and the emergence of Nipah virus: a lethal bat‐borne zoonosis. Journal of the Royal Society Interface 9: 89–101. 21632614 10.1098/rsif.2011.0223 PMC3223631

[mam12201-bib-0090] Rajala EL , Sattorov N , Boqvist S , Magnusson U (2017) Bovine leptospirosis in urban and peri‐urban dairy farming in low‐income countries: a “One Health” issue? Acta Veterinaria Scandinavica 59: 83. 29233193 10.1186/s13028-017-0352-6 PMC5727926

[mam12201-bib-0091] Regoes RR , Hamblin S , Tanaka MM (2012) Viral mutation rates: modelling the roles of within‐host viral dynamics and the trade‐off between replication fidelity and speed. Proceedings of the Royal Society B: Biological Sciences 280: 20122047. 10.1098/rspb.2012.2047 PMC3574426 23135674

[mam12201-bib-0092] Rey J , Lounibos L , Padmanabha H , Mosquera M (2010) Emergence of dengue fever in America: patterns, processes and prospects. Interciencia 35: 800–806.

[mam12201-bib-0093] Rondon S , Ortiz M , Leon C , Galvis N , Link A , Gonzalez C (2017) Seasonality, richness and prevalence of intestinal parasites of three Neotropical primates (*Alouatta seniculus*, *Ateles hybridus* and *Cebus versicolor*) in a fragmented forest in Colombia. International Journal for Parasitology‐Parasites and Wildlife 6: 202–208. 28794984 10.1016/j.ijppaw.2017.07.006 PMC5537371

[mam12201-bib-0094] Rothenburger JL , Himsworth CG , Nemeth NM , Pearl DL , Jardine CM (2018) Beyond abundance: how microenvironmental features and weather influence *Bartonella tribocorum* infection in wild Norway rats (*Rattus norvegicus*). Zoonoses and Public Health 65: 339–351. 29274119 10.1111/zph.12440

[mam12201-bib-0095] Salyer SJ , Gillespie TR , Rwego IB , Chapman CA , Goldberg TL (2012) Epidemiology and molecular relationships of *Cryptosporidium* spp. in people, primates, and livestock from Western Uganda. PLoS Neglected Tropical Diseases 6: e1597. 22506085 10.1371/journal.pntd.0001597 PMC3323507

[mam12201-bib-0096] Sato S , Tojo B , Hoshi T , Minsong LIF , Kugan OK , Giloi N et al. (2019) Recent incidence of human malaria caused by *Plasmodium knowlesi* in the villages in Kudat Peninsula, Sabah, Malaysia: mapping of the infection risk using remote sensing data. International Journal of Environmental Research and Public Health 16: E2954. 31426380 10.3390/ijerph16162954 PMC6720544

[mam12201-bib-0097] Scinachi CA , Takeda GACG , Mucci LF , Pinter A (2017) Association of the occurrence of Brazilian spotted fever and Atlantic rain forest fragmentation in the São Paulo metropolitan region, Brazil. Acta Tropica 166: 225–233. 27880877 10.1016/j.actatropica.2016.11.025

[mam12201-bib-0098] Sehgal RNM (2010) Deforestation and avian infectious diseases. The Journal of Experimental Biology 213: 955–960. 20190120 10.1242/jeb.037663 PMC2829318

[mam12201-bib-0099] Shapiro JT , da Costa Lima Junior MS , Dorval MEC , de Oliveira França A , Cepa Matos MF , Bordignon MO (2013) First record of *Leishmania braziliensis* presence detected in bats, Mato Grosso do Sul, southwest Brazil. Acta Tropica 128: 171–174. 23886850 10.1016/j.actatropica.2013.07.004

[mam12201-bib-0100] Sharma VP , Kondrashin A . (1991) Forest malaria in Southeast Asia. *Proceedings of an Informal Consultative Meeting, WHO/MRC*, 234.

[mam12201-bib-0101] Smit LAM , Heederik D (2017) Impacts of intensive livestock production on human health in densely populated regions. GeoHealth 1: 272–277. 32158992 10.1002/2017GH000103 PMC7007140

[mam12201-bib-0102] Springer A , Montenegro VM , Schicht S , Vrohvec MG , Pantchev N , Balzer J , Strube C (2019) Seroprevalence and current infections of canine vector‐borne diseases in Costa Rica. Frontiers in Veterinary Science 6: 164. 31214605 10.3389/fvets.2019.00164 PMC6558105

[mam12201-bib-0103] Swei A , Couper LI , Coffey LL , Kapan D , Bennett S (2020) Patterns, drivers, and challenges of vector‐borne disease emergence. Vector‐Borne and Zoonotic Diseases 20: 159–170. 31800374 10.1089/vbz.2018.2432 PMC7640753

[mam12201-bib-0104] Taku A , Bhat M , Dutta T , Chhabra R (2007) Viral diseases transmissible from non‐human primates to man. Indian Journal of Virology 18: 47–56.

[mam12201-bib-0105] Taylor D (1997) Seeing the forests for more than the trees. Environmental Health Perspectives 105: 1186. 9370516 10.1289/ehp.971051186 PMC1470324

[mam12201-bib-0106] Taylor LH , Latham SM , Woolhouse MEJ (2001) Risk factors for human disease emergence. Philosophical Transactions of the Royal Society of London. Series B: Biological Sciences 356: 983–989. 11516376 10.1098/rstb.2001.0888 PMC1088493

[mam12201-bib-0107] Thatcher HR , Downs CT , Koyama NF (2018) Using parasitic load to measure the effect of anthropogenic disturbance on vervet monkeys. EcoHealth 15: 676–681. 30091030 10.1007/s10393-018-1349-y PMC6245093

[mam12201-bib-0108] Thompson RCA (2000) Giardiasis as a re‐emerging infectious disease and its zoonotic potential. International Journal for Parasitology 30: 1259–1267. 11113253 10.1016/s0020-7519(00)00127-2

[mam12201-bib-0109] Tilman D , Cassman KG , Matson PA , Naylor R , Polasky S (2002) Agricultural sustainability and intensive production practices. Nature 418: 671–677. 12167873 10.1038/nature01014

[mam12201-bib-0110] Tomley FM , Shirley MW (2009) Livestock infectious diseases and zoonoses. Philosophical Transactions of the Royal Society B: Biological Sciences 364: 2637–2642. 10.1098/rstb.2009.0133 PMC2865087 19687034

[mam12201-bib-0111] Twiddy SS , Holmes EC , Rambaut A (2003) Inferring the rate and time‐scale of dengue virus evolution. Molecular Biology and Evolution 20: 122–129. 12519914 10.1093/molbev/msg010

[mam12201-bib-0112] Umhang G , Comte S , Raton V , Hormaz V , Boucher J‐M , Favier S , Combes B , Boué F (2014) *Echinococcus multilocularis* infections in dogs from urban and peri‐urban areas in France. Parasitology Research 113: 2219–2222. 24687286 10.1007/s00436-014-3875-z

[mam12201-bib-0113] United Nations (2016) The World's Cities in 2016. https://www.un.org/en/development/desa/population/publications/pdf/urbanization/the_worlds_cities_in_2016_data_booklet.pdf [accessed January 2019].

[mam12201-bib-0114] Walker M , Wilcox B , Wong M (2008) Waterborne zoonoses and changes in hydrologic response due to watershed development. In: Fares A , El‐Kadi AI (eds) Coastal Watershed Management 349–367. WIT Press, Southampton, UK

[mam12201-bib-0115] WHO (2019) Disease outbreaks by year. www.who.int/csr/don/archive/year/en/ [accessed 25 November 2019].

[mam12201-bib-0116] Wild TF (2009) Henipaviruses: a new family of emerging paramyxoviruses. Pathologie Biologie 57: 188–196. 18511217 10.1016/j.patbio.2008.04.006

[mam12201-bib-0117] Willig MR , Presley SJ , Plante JL , Bloch CP , Solari S , Pacheco V , Weaver SC (2019) Guild‐level responses of bats to habitat conversion in a lowland Amazonian rainforest: species composition and biodiversity. Journal of Mammalogy 100: 223–238. 30846887 10.1093/jmammal/gyz023 PMC6394116

[mam12201-bib-0118] Woolhouse MEJ , Gowtage‐Sequeria S (2005) Host range and emerging and reemerging pathogens. Emerging Infectious Diseases 11: 1842–1847. 16485468 10.3201/eid1112.050997 PMC3367654

[mam12201-bib-0119] Xie Y , Hoberg EP , Yang Z , Urban JF , Yang G (2017) *Ancylostoma ailuropodae* n. spp. (Nematoda: Ancylostomatidae), a new hookworm parasite isolated from wild giant pandas in Southwest China. Parasites & Vectors 10: 277. 28576124 10.1186/s13071-017-2209-2 PMC5457663

[mam12201-bib-0120] Zhang Y‐Z , Holmes EC (2020) A genomic perspective on the origin and emergence of SARS‐CoV‐2. Cell 181: 223–227. 32220310 10.1016/j.cell.2020.03.035 PMC7194821

