# Supplementary material for: Emerging zoonotic diseases originating in mammals: a systematic review of effects of anthropogenic land‐use change
Source: Mamm Rev. 2020 Jun 2;50(4):336–52. doi: 10.1111/mam.12201 (PMC7300897; doi:10.1111/mam.12201)
Supplement: Supplementary file 1 — Appendix S1. List of papers obtained from the systematic review search string. [file MAM-50-336-s001.docx]

**Appendix S1 – List of papers obtained from the systematic review search string**

White RJ, Razgour O (2020) Emerging zoonotic diseases originating in mammals: a systematic review of effects of anthropogenic land-use change**.** *Mammal Review*

1. Anders JL, Nakao M, Uchida K, Ayer CG, Asakawa M, Koizumi I (2019) Comparison of the intestinal helminth community of the large Japanese field mouse (Apodemus speciosus) between urban, rural, and natural sites in Hokkaido, Japan. *Parasitology International* 70: 51–57.
2. Barthod C, Fournier P (2019) Forests and health: discourse and practises from the 18st to the 21st century. *Sante Publique* 31: 15–23.
3. Blasdell KR, Morand S, Perera D, Firth C (2019) Association of rodent-borne Leptospira spp. with urban environments in Malaysian Borneo. *Plos Neglected Tropical Diseases* 13: e0007141.
4. Borremans B, Faust C, Manlove KR, Sokolow SH, Lloyd-Smith JO (2019) Cross-species pathogen spillover across ecosystem boundaries: mechanisms and theory. *Philosophical Transactions of the Royal Society B-Biological Sciences* 374: 20180344.
5. Brock PM, Fornace KM, Grigg MJ, Anstey NM, William T, Cox J, Drakeley CJ, Ferguson HM, Kao RR (2019) Predictive analysis across spatial scales links zoonotic malaria to deforestation. *Proceedings of the Royal Society B-Biological Sciences* 286: 20182351.
6. Chauhan AS, George MS, Lindahl J, Grace D, Kakkar M (2019) Community, system and policy level drivers of bovine tuberculosis in smallholder periurban dairy farms in India: a qualitative enquiry. *BMC Public Health* 19: 301.
7. Combe M, Gozlan RE, Jagadesh S, Velvin CJ, Ruffine R, Demar MP et al. (2019) Comparison of Mycobacterium ulcerans (Buruli ulcer) and Leptospira sp. (Leptospirosis) dynamics in urban and rural settings. *PLOS Neglected Tropical Diseases* 13: e0007074.
8. Davidson G, Chua TH, Cook A, Speldewinde P, Weinstein P (2019) Defining the ecological and evolutionary drivers of Plasmodium knowlesi transmission within a multi-scale framework. *Malaria Journal* 18: 66.
9. Dingle KE, Didelot X, Quan TP, Eyre DW, Stoesser N, Marwick CA et al. (2019) A Role for Tetracycline Selection in Recent Evolution of Agriculture-Associated Clostridium difficile PCR Ribotype 078. *Mbio* 10: e02790-18.
10. Fernandes J, de Oliveira RC, Coelho TA, Martins RMB, Caetano KAA, Horta MAP et al. (2019) Rodent-borne viruses survey in rural settlers from Central Brazil. *Memorias Do Instituto Oswaldo Cruz* 114: e180448.
11. Guegan JF, de Thoisy B, Ayouba A, Cappelle J (2019) Tropical forests, changes in land uses and emerging infectious hazards. *Sante Publique* 31: 91–106.
12. Hassell JM, Ward MJ, Muloi D, Bettridge JM, Phan H, Robinson TP et al. (2019) Deterministic processes structure bacterial genetic communities across an urban landscape. *Nature Communications* 10: 2643.
13. Hawkes FM, Manin BO, Cooper A, Daim S, Homathevi R, Jelip J, Husin T, Chua TH (2019) Vector compositions change across forested to deforested ecotones in emerging areas of zoonotic malaria transmission in Malaysia. *Scientific Reports* 9: 13312.
14. Heylen D, Lasters R, Adriaensen F, Fonville M, Sprong H, Matthysen E (2019) Ticks and tick-borne diseases in the city: Role of landscape connectivity and green space characteristics in a metropolitan area. *Science of the Total Environment* 670: 941–949.
15. Hillman AE, Ash AL, Lymbery AJ, Thompson RCA (2019) Anthropozoonotic significance, risk factors and spatial distribution of Giardia spp. infections in quenda (Isoodon obesulus) in the greater Perth region, Western Australia. *International Journal for Parasitology-Parasites and Wildlife* 9: 42–48.
16. Hunsperger E, Juma B, Onyango C, Ochieng JB, Omballa V, Fields BS et al. (2019) Building laboratory capacity to detect and characterize pathogens of public and global health security concern in Kenya. *BMC Public Health* 19: 477.
17. Jnov E (2019) Emerging and threatening vector-borne zoonoses in the world and in Europe: a brief update. *Pathogens and Global Health* 113: 49–57.
18. Loaiza JR, Rovira JR, Sanjur OI, Zepeda JA, Pecor JE, Foley DH et al. (2019) Forest disturbance and vector transmitted diseases in the lowland tropical rainforest of central Panama. *Tropical Medicine & International Health* 24: 849–861.
19. MacDonald AJ, Larsen AE, Plantinga AJ (2019) Missing the people for the trees: Identifying coupled natural-human system feedbacks driving the ecology of Lyme disease. *Journal of Applied Ecology* 56: 354–364.
20. Majewska AA, Satterfield DA, Harrison RB, Altizer S, Hepinstall-Cymerman J (2019) Urbanization predicts infection risk by a protozoan parasite in non-migratory populations of monarch butterflies from the southern coastal US and Hawaii. *Landscape Ecology* 34: 649–661.
21. Martinez-de la Puente J, Ferraguti M, Jimenez-Penuela J, Ruiz S, Martinez J, Roiz D, Soriguer R, Figuerola J (2019) Filarial worm circulation by mosquitoes along an urbanization gradient in southern Spain. *Transboundary and Emerging Diseases* 66: 1752–1757.
22. Morand S, Blasdell K, Bordes F, Buchy P, Carcy B, Chaisiri K et al. (2019) Changing landscapes of Southeast Asia and rodent-borne diseases: decreased diversity but increased transmission risks. *Ecological Applications* 29: e01886.
23. Munyua PM, Njenga MK, Osoro EM, Onyango CO, Bitek AO, Mwatondo A et al. (2019) Successes and challenges of the One Health approach in Kenya over the last decade. *BMC Public Health* 19: 465.
24. Nantima N, Ilukor J, Kaboyo W, Ademun ARO, Muwanguzi D, Sekamatte M, Sentumbwe J, Monje F, Bwire G (2019) The importance of a One Health approach for prioritising zoonotic diseases to focus on capacity-building efforts in Uganda. *Revue Scientifique Et Technique-Office International Des Epizooties* 38: 315–325.
25. Narciso TP, Carvalho RC, Campos LC, Viana AG, Fujiwara RT, Barcante TA, Alvarenga IM, Barcante JMD (2019) First report of an autochthonous human visceral leishmaniasis in a child from the South of Minas Gerais State, Brazil. *Revista Do Instituto De Medicina Tropical De Sao Paulo* 61: e1.
26. Newton EJ, Pond BA, Tinline RR, Middel K, Belanger D, Rees EE (2019) Differential impacts of vaccination on wildlife disease spread during epizootic and enzootic phases. *Journal of Applied Ecology* 56: 526–536.
27. Nguyen HTT, Afriyie DO, Tran CH, Dang AD, Tran DN, Dang TO et al. (2019) Progress towards rabies control and elimination in Vietnam. *Revue Scientifique Et Technique-Office International Des Epizooties* 38: 199–212
28. .Otranto D, Deplazes P (2019) Zoonotic nematodes of wild carnivores. *International Journal for Parasitology-Parasites and Wildlife* 9: 370–383.
29. Pizarro-Araya J, Alfaro FM, Munoz-Rivera RA, Barriga-Tunon JE, Letelier L (2019) Arthropods of forestry and medical-veterinary importance in the Limari basin (Coquimbo region, Chile). *Ciencia E Investigacion Agraria* 46: 40–49.
30. Rizzoli A, Tagliapietra V, Cagnacci F, Marini G, Arnoldi D, Rosso F, Rosa R (2019) Parasites and wildlife in a changing world: The vector-host- pathogen interaction as a learning case. *International Journal for Parasitology-Parasites and Wildlife* 9: 394–401.
31. Rohrl JR, Barrett CB, Civitello DJ, Craft ME, Delius B, DeLeo GA et al. (2019) Emerging human infectious diseases and the links to global food production. *Nature Sustainability* 2: 445–456.
32. Roldan JS, Candurra WA, Colombo MI, Delgui LR (2019) Junin virus promotes autophagy to facilitate the virus life cycle. *Journal of Virology* 93: e02307-18.
33. Sato S, Tojo B, Hoshi T, Minsong LIF, Kugan OK, Giloi N et al. (2019) Recent incidence of human malaria caused by Plasmodium knowlesi in the villages in Kudat Peninsula, Sabah, Malaysia: mapping of the infection risk using remote sensing data. *International Journal of Environmental Research and Public Health* 16: E2954.
34. Springer A, Montenegro VM, Schicht S, Vrohvec MG, Pantchev N, Balzer J, Strube C (2019) Seroprevalence and Current Infections of Canine Vector-Borne Diseases in Costa Rica. *Frontiers in Veterinary Science* 6: 164.
35. Sukhralia S, Verma M, Gopirajan S, Dhanaraj PS, Lal R, Mehla N, Kant CR (2019) From dengue to Zika: the wide spread of mosquito-borne arboviruses. *European Journal of Clinical Microbiology & Infectious Diseases* 38: 3–14.
36. Tryland M, Nymo IH, Romano JS, Mork T, Klein J, Rockstrom U (2019) Infectious disease outbreak associated with supplementary feeding of semi-domesticated reindeer. *Frontiers in Veterinary Science* 6: 126.
37. Walsh MG (2019) Ecological and life history traits are associated with Ross River virus infection among sylvatic mammals in Australia. *Bmc Ecology* 19: 2.
38. Wang L, Zou Y, Zhu XP, Bottazzi ME, Hotez PJ, Zhan B (2019) China’s shifting neglected parasitic infections in an era of economic reform, urbanization, disease control, and the Belt and Road Initiative. *Plos Neglected Tropical Diseases* 13: e0006946.
39. Willig MR, Presley SJ, Plante JL, Bloch CP, Solari S, Pacheco V, Weaver SC (2019) Guild-level responses of bats to habitat conversion in a lowland Amazonian rainforest: species composition and biodiversity. *Journal of Mammalogy* 100: 223–238.
40. Adalsteinsson SA, Shriver WG, Hojgaard A, Bowman JL, Brisson D, D’Amico V, Buler JJ (2018) Multiflora rose invasion amplifies prevalence of Lyme disease pathogen, but not necessarily Lyme disease risk. *Parasites & Vectors* 11: 54.
41. Afelt A, Lacroix A, Zawadzka-Pawlewska U, Pokojski W, Buchy P, Frutos R (2018) Distribution of bat-borne viruses and environment patterns. *Infection Genetics and Evolution* 58: 181–191.
42. Becker DJ, Teitelbaum CS, Murray MH, Curry SE, Welch CN, Ellison T et al. (2018) Assessing the contributions of intraspecific and environmental sources of infection in urban wildlife: Salmonella enterica and white ibis as a case study. *Journal of The Royal Society Interface* 15: 20180654.
43. Brown LM, Hall RJ (2018) Consequences of resource supplementation for disease risk in a partially migratory population. *Philosophical Transactions of the Royal Society B: Biological Sciences* 373: 20170095.
44. Brown R, Hing CT, Fornace K, Ferguson HM (2018) Evaluation of resting traps to examine the behaviour and ecology of mosquito vectors in an area of rapidly changing land use in Sabah, Malaysian Borneo. *Parasites & Vectors* 11: 346.
45. Chung DM, Ferree E, Simon DM, Yeh PJ (2018) Patterns of bird–bacteria associations. *EcoHealth* 15: 627–641.
46. Clark NJ, Soares Magalhães RJ (2018) Airborne geographical dispersal of Q fever from livestock holdings to human communities: a systematic review and critical appraisal of evidence. *BMC Infectious Diseases* 18: 218.
47. Cortez V, Canal E, Dupont-Turkowsky JC, Quevedo T, Albujar C, Chang T-C et al. (2018) Identification of Leptospira and Bartonella among rodents collected across a habitat disturbance gradient along the Inter-Oceanic Highway in the southern Amazon Basin of Peru. *PLOS ONE* 13: e0205068.
48. Craighead L, Meyer A, Chengat B, Musallam I, Akakpo J, Kone P, Guitian J, Häsler B (2018) Brucellosis in West and Central Africa: A review of the current situation in a changing landscape of dairy cattle systems. *Acta Tropica* 179: 96–108.
49. Dhimal M, Dahal S, Dhimal ML, Mishra SR, Karki KB, Aryal KK et al. (2018) Threats of Zika virus transmission for Asia and its Hindu-Kush Himalayan region. *Infectious Diseases of Poverty* 7: 87.
50. Eskew EA, Olival KJ (2018) De-urbanization and zoonotic disease risk. *EcoHealth* 15: 707–712.
51. Fatima SH, Zaidi F, Adnan M, Ali A, Jamal Q, Khisroon M (2018) Rat-bites of an epidemic proportion in Peshawar vale; a GIS based approach in risk assessment. *Environmental Monitoring and Assessment* 190: 233.
52. Fedeli C, Moreno H, Kunz S (2018) Novel insights into cell entry of emerging human pathogenic arenaviruses. *Journal of Molecular Biology* 430: 1839–1852.
53. Glennon EE, Restif O, Sbarbaro SR, Garnier R, Cunningham AA, Suu-Ire RD, Osei-Amponsah R, Wood JLN, Peel AJ (2018) Domesticated animals as hosts of henipaviruses and filoviruses: A systematic review. *The Veterinary Journal* 233: 25–34.
54. Hancke D, Suarez OV (2018) Structure of parasite communities in urban environments: the case of helminths in synanthropic rodents. *Folia Parasitologica* 65.
55. Kuiken T, Breitbart M, Beer M, Grund C, Höper D, van den Hoogen B et al. (2018) Zoonotic infection with pigeon paramyxovirus type 1 linked to fatal pneumonia. *The Journal of Infectious Diseases* 218: 1037–1044.
56. Kurucz K, Madai M, Bali D, Hederics D, Horváth G, Kemenesi G, Jakab F (2018) Parallel survey of two widespread renal syndrome-causing zoonoses: Leptospira spp. and hantavirus in urban environment, Hungary. *Vector-Borne and Zoonotic Diseases* 18: 200–205.
57. Leite BMM, Solcà M da S, Santos LCS, Coelho LB, Amorim LDAF, Donato LE et al. (2018) The mass use of deltamethrin collars to control and prevent canine visceral leishmaniasis: A field effectiveness study in a highly endemic area. *PLOS Neglected Tropical Diseases* 12: e0006496.
58. Linske MA, Williams SC, Stafford KC, Ortega IM (2018) Ixodes scapularis (Acari: Ixodidae) reservoir host diversity and abundance impacts on dilution of Borrelia burgdorferi (Spirochaetales: Spirochaetaceae) in residential and woodland habitats in Connecticut, United States. *Journal of Medical Entomology* 55: 681–690.
59. Maaz D, Krücken J, Blümke J, Richter D, McKay-Demeler J, Matuschka F-R, Hartmann S, von Samson-Himmelstjerna G (2018) Factors associated with diversity, quantity and zoonotic potential of ectoparasites on urban mice and voles. *PLOS ONE* 13: e0199385.
60. MacDonald AJ, Hyon DW, McDaniels A, O’Connor KE, Swei A, Briggs CJ (2018) Risk of vector tick exposure initially increases, then declines through time in response to wildfire in California. *Ecosphere* 9(5):e02227.
61. McMahon BJ, Morand S, Gray JS (2018) Ecosystem change and zoonoses in the Anthropocene. *Zoonoses and Public Health* 65: 755–765.
62. Millins C, Dickinson ER, Isakovic P, Gilbert L, Wojciechowska A, Paterson V et al. (2018) Landscape structure affects the prevalence and distribution of a tick-borne zoonotic pathogen. *Parasites & Vectors* 11: 621.
63. Miro G, Lopez-Velez R (2018) Clinical management of canine leishmaniosis versus human leishmaniasis due to Leishmania infantum: Putting “One Health” principles into practice. *Veterinary Parasitology* 254: 151–159.
64. Neves ES, Mendenhall IH, Borthwick SA, Su YCF, Smith GJD (2018) Detection and genetic characterization of diverse Bartonella genotypes in the small mammals of Singapore. *Zoonoses and Public Health* 65: E207–E215.
65. Pech-May A, Ramsey JM, González Ittig RE, Giuliani M, Berrozpe P, Quintana MG, Salomón OD (2018) Genetic diversity, phylogeography and molecular clock of the Lutzomyia longipalpis complex (Diptera: Psychodidae). *PLOS Neglected Tropical Diseases* 12: e0006614.
66. Perez-Rodriguez A, Khimoun A, Ollivier A, Eraud C, Faivre B, Garnier S (2018) Habitat fragmentation, not habitat loss, drives the prevalence of blood parasites in a Caribbean passerine. *Ecography* 41: 1835–1849.
67. Picoloto C, do Nascimento VF, Hattori TY, Melo AVG, da Silva JH, dos Santos RAN, Tercas-Trettel ACP (2018) Clinical-epidemiological aspects of convalescent patients after hantavirus infection in Mato Grosso. *Revista De Epidemiologia E Controle De Infeccao* 8.
68. Restrepo AMC, Yang YR, McManus DP, Gray DJ, Barnes TS, Williams GM, Magalhaes RJS, Hamm NAS, Clements ACA (2018) Spatiotemporal patterns and environmental drivers of human echinococcoses over a twenty-year period in Ningxia Hui Autonomous Region, China. *Parasites & Vectors* 11: 108.
69. Riesle-Sbarbaro SA, Amponsah-Mensah K, de Vries S, Nicolas V, Lalis A, Suu-Ire R, Cunningham AA, Wood JLN, Sargan DR (2018) The Gambian epauletted fruit bat shows increased genetic divergence in the Ethiopian highlands and in an area of rapid urbanization. *Ecology and Evolution* 8: 12803–12820.
70. Rodrigues TCS, Diaz-Delgado J, Catao-Dias JL, Carvalho JD, Marmontel M (2018) Retrospective pathological survey of pulmonary disease in free-ranging Amazon river dolphin Inia geoffrensis and tucuxi Sotalia fluviatilis. *Diseases of Aquatic Organisms* 131: 1–11.
71. Rothenburger JL, Himsworth CG, Nemeth NM, Pearl DL, Jardine CM (2018) Beyond abundance: How microenvironmental features and weather influence Bartonella tribocorum infection in wild Norway rats (Rattus norvegicus). *Zoonoses and Public Health* 65: 339–351.
72. Salerno J, Chapman CA, Diem JE, Dowhaniuk N, Goldman A, MacKenzie CA et al. (2018) Park isolation in anthropogenic landscapes: land change and livelihoods at park boundaries in the African Albertine Rift. *Regional Environmental Change* 18: 913–928.
73. Santovito E, Greco D, Logrieco AF, Avantaggiato G (2018) Eubiotics for Food Security at Farm Level: Yeast Cell Wall Products and Their Antimicrobial Potential Against Pathogenic Bacteria. *Foodborne Pathogens and Disease* 15: 531–537.
74. Schnyder M, Hertzberg H, Mathis A, Schonmann M, Hehl A, Deplazes P (2018) Veterinary parasitology teaching Ten years of experience with the Vetsuisse curriculum. *Veterinary Parasitology* 252: 148–152.
75. Scott DM, Baker R, Charman N, Karlsson H, Yarnell RW, Mill AC, Smith GC, Tolhurst BA (2018) A citizen science based survey method for estimating the density of urban carnivores. *PLOS ONE* 13: e0197445.
76. Shah V, Shah A, Joshi V (2018) Predicting the origins of next forest-based emerging infectious disease. *Environmental Monitoring and Assessment* 190: 337.
77. Supramaniam A, Lui H, Bellette BM, Rudd PA, Herrero LJ (2018) How myeloid cells contribute to the pathogenesis of prominent emerging zoonotic diseases. *Journal of General Virology* 99: 953–969.
78. Thatcher HR, Downs CT, Koyama NF (2018) Using parasitic load to measure the effect of anthropogenic disturbance on vervet monkeys. *EcoHealth* 15: 676–681.
79. Tian H, Hu S, Cazelles B, Chowell G, Gao L, Laine M et al. (2018) Urbanization prolongs hantavirus epidemics in cities. *Proceedings of the National Academy of Sciences* 115: 4707–4712.
80. Tomassone L, Berriatua E, De Sousa R, Duscher GG, Mihalca AD, Silaghi C, Sprong H, Zintl A (2018) Neglected vector-borne zoonoses in Europe: Into the wild. *Veterinary Parasitology* 251: 17–26.
81. Trinh P, Zaneveld JR, Safranek S, Rabinowitz PM (2018) One Health relationships between human, animal, and environmental microbiomes: a mini-review. *Frontiers in Public Health* 6: 235
82. Vogeler AVB, Tschapka M, Kalko EK V, Cottontail VM (2018) Litomosoides microfilaria in seven Neotropical bat species. *Journal of Parasitology* 104: 713–717.
83. Vythilingam I, Wong ML, Wan-Yussof WS (2018) Current status of Plasmodium knowlesi vectors: a public health concern? *Parasitology* 145: 32–40.
84. Wilkinson DA, Marshall JC, French NP, Hayman DTS (2018) Habitat fragmentation, biodiversity loss and the risk of novel infectious disease emergence. *Journal of the Royal Society Interface* 15: 20180403.
85. Abuzaid AA, Abdoon AM, Aldahan MA, Alzahrani AG, Alhakeem RF, Asiri AM, Alzahrani MH, Memish ZA (2017) Cutaneous leishmaniasis in Saudi Arabia: a comprehensive overview. *Vector-Borne and Zoonotic Diseases* 17: 673–684.
86. Andersen SC, Fachmann MSR, Kiil K, Nielsen EM, Hoorfar J (2017) Gene-based pathogen detection: can we use qPCR to predict the outcome of diagnostic metagenomics? *Genes* 8: 332.
87. Arthur RF, Gurley ES, Salje H, Bloomfield LSP, Jones JH (2017) Contact structure, mobility, environmental impact and behaviour: the importance of social forces to infectious disease dynamics and disease ecology. *Philosophical Transactions of the Royal Society B: Biological Sciences* 372: 20160454.
88. Bardosh KL, Scoones JC, Grace D, Kalema-Zikusoka G, Jones KE, de Balogh K et al. (2017) Engaging research with policy and action: what are the challenges of responding to zoonotic disease in Africa? *Philosophical Transactions of the Royal Society B: Biological Sciences* 372: 20160172.
89. Caldart ET, Freire RL, Ferreira FP, Ruffolo BB, Sbeghen MR, Mareze M, Garcia JL, Mitsuka-Breganó R, Navarro IT (2017) Leishmania in synanthropic rodents (Rattus rattus): new evidence for the urbanization of Leishmania (Leishmania) amazonensis. *Revista Brasileira de Parasitologia Veterinária* 26: 17–27.
90. Calle AI, Mari RB, de las Heras E, Lucientes J, Molina R (2017) Climate Change in Spain and its Influence on Vector-Transmitted Diseases. *Revista De Salud Ambiental* 17: 70–86.
91. Donalisio MR, Paiz LM, da Silva VG, Richini-Pereira VB, von Zuben APB, Castagna CL, Motoie G, Hiramoto RM, Tolezano JE (2017) Visceral leishmaniasis in an environmentally protected area in southeastern Brazil: Epidemiological and laboratory cross-sectional investigation of phlebotomine fauna, wild hosts and canine cases. *PLOS Neglected Tropical Diseases* 11: e0005666.
92. Galvis-Ovallos F, Casanova C, Sevá A da P, Galati EAB (2017) Ecological parameters of the (S)-9-methylgermacrene-B population of the Lutzomyia longipalpis complex in a visceral leishmaniasis area in São Paulo state, Brazil. *Parasites & Vectors* 10: 269.
93. Hansen A, Xiang J, Liu Q, Tong MX, Sun Y, Liu X et al. (2017) Experts’ perceptions on China’s capacity to manage emerging and re-emerging zoonotic diseases in an era of climate change. *Zoonoses and Public Health* 64: 527–536.
94. Hassell JM, Begon M, Ward MJ, Fèvre EM (2017) Urbanization and disease emergence: dynamics at the wildlife–livelstock–human interface. *Trends in Ecology & Evolution* 32: 55–67.
95. Hillman AE, Lymbery AJ, Elliot AD, Andrew Thompson RC (2017) Urban environments alter parasite fauna, weight and reproductive activity in the quenda (Isoodon obesulus). *Science of The Total Environment* 607–608: 1466–1478.
96. Hosseini PR, Mills JN, Prieur-Richard A-H, Ezenwa VO, Bailly X, Rizzoli A et al. (2017) Does the impact of biodiversity differ between emerging and endemic pathogens? The need to separate the concepts of hazard and risk. *Philosophical Transactions of the Royal Society B: Biological Sciences* 372: 20160129.
97. Hu WL, Dong HY, Li Y, Ojcius DM, Li SJ, Yan J (2017) Bid-induced release of AIF/EndoG from mitochondria causes apoptosis of macrophages during Infection with Leptospira interrogans. *Frontiers in Cellular and Infection Microbiology* 7: 471.
98. Kilpatrick AM, Dobson ADM, Levi T, Salkeld DJ, Swei A, Ginsberg HS et al. (2017) Lyme disease ecology in a changing world: consensus, uncertainty and critical gaps for improving control. *Philosophical Transactions of the Royal Society B-Biological Sciences* 372: 20160117.
99. Kilpatrick AM, Salkeld DJ, Titcomb G, Hahn MB (2017) Conservation of biodiversity as a strategy for improving human health and well-being. *Philosophical Transactions of the Royal Society B: Biological Sciences* 372: 20160131.
100. Klaus A, Zimmermann E, Roper KM, Radespiel U, Nathan S, Goossens B, Strube C (2017) Co-infection patterns of intestinal parasites in arboreal primates (proboscis monkeys, Nasalis larvatus) in Borneo. *International Journal for Parasitology-Parasites and Wildlife* 6: 320–329.
101. Layton DS, Choudhary A, Bean AGD (2017) Breaking the chain of zoonoses through biosecurity in livestock. *Vaccine* 35: 5967–5973.
102. Millins C, Gilbert L, Medlock J, Hansford K, Thompson DBA, Biek R (2017) Effects of conservation management of landscapes and vertebrate communities on Lyme borreliosis risk in the United Kingdom. *Philosophical Transactions of the Royal Society B: Biological Sciences* 372: 20160123.
103. Motta P, Porphyre T, Handel I, Hamman SM, Ngu Ngwa V, Tanya V, Morgan K, Christley R, Bronsvoort BM deC. (2017) Implications of the cattle trade network in Cameroon for regional disease prevention and control. *Scientific Reports* 7: 43932.
104. Nava A, Shimabukuro JS, Chmura AA, Luz SLB (2017) The impact of global environmental changes on infectious disease emergence with a focus on risks for Brazil. *ILAR Journal* 58: 393–400.
105. Nii-Trebi NI (2017) Emerging and neglected infectious diseases: insights, advances, and challenges. *Biomed Research International*: 1–15.
106. Nunes H, Rocha FL, Cordeiro-Estrela P (2017) Bats in urban areas of Brazil: roosts, food resources and parasites in disturbed environments. *Urban Ecosystems* 20: 953–969.
107. Otero-Abad B, Rüegg SR, Hegglin D, Deplazes P, Torgerson PR (2017) Mathematical modelling of Echinococcus multilocularis abundance in foxes in Zurich, Switzerland. *Parasites & Vectors* 10: 21.
108. Patil RR, Kumar CS, Bagvandas M (2017) Biodiversity loss: Public health risk of disease spread and epidemics. *Annals of Tropical Medicine and Public Health* 10: 1432–1438.
109. Peterson AC, Ghersi BM, Alda F, Firth C, Frye MJ, Bai Y et al. (2017) Rodent-borne bartonella infection varies according to host species within and among cities. *EcoHealth* 14: 771–782.
110. Pollack L, Ondrasek NR, Calisi R (2017) Urban health and ecology: the promise of an avian biomonitoring tool. *Current Zoology* 63: 205–212.
111. Rajala EL, Sattorov N, Boqvist S, Magnusson U (2017) Bovine leptospirosis in urban and peri-urban dairy farming in low-income countries: a “One Health” issue? *Acta Veterinaria Scandinavica* 59: 83.
112. Richardson JL, Burak MK, Hernandez C, Shirvell JM, Mariani C, Carvalho-Pereira TSA et al. (2017) Using fine-scale spatial genetics of Norway rats to improve control efforts and reduce leptospirosis risk in urban slum environments. *Evolutionary Applications* 10: 323–337.
113. Rondon S, Ortiz M, Leon C, Galvis N, Link A, Gonzalez C (2017) Seasonality, richness and prevalence of intestinal parasites of three neotropical primates (Alouatta seniculus, Ateles hybridus and Cebus versicolor) in a fragmented forest in Colombia. *International Journal for Parasitology-Parasites and Wildlife* 6: 202–208.
114. Rulli MC, Santini M, Hayman DTS, D’Odorico P (2017) The nexus between forest fragmentation in Africa and Ebola virus disease outbreaks. *Scientific Reports* 7: 41613.
115. Salerno J, Ross N, Ghai R, Mahero M, Travis DA, Gillespie TR, Hartter J (2017) Human-Wildlife Interactions Predict Febrile Illness in Park Landscapes of Western Uganda. *Ecohealth* 14: 675–690.
116. Scinachi CA, Takeda GACG, Mucci LF, Pinter A (2017) Association of the occurrence of Brazilian spotted fever and Atlantic rain forest fragmentation in the São Paulo metropolitan region, Brazil. *Acta Tropica* 166: 225–233.
117. Smit LAM, Heederik D (2017) Impacts of Intensive Livestock Production on Human Health in Densely Populated Regions. *GeoHealth* 1: 272–277.
118. Toi CS, Webb CE, Haniotis J, Clancy J, Doggett SL (2017) Seasonal activity, vector relationships and genetic analysis of mosquito-borne Stratford virus. *PLOS ONE* 12: e0173105.
119. Walsh MG, Wiethoelter A, Haseeb MA (2017) The impact of human population pressure on flying fox niches and the potential consequences for Hendra virus spillover. *Scientific Reports* 7: 8226.
120. Wasserberg G, Smyth C, Tsurim I (2017) The role of anthropogenic land-use change in driving disease emergence in highly-coupled vector-host systems: Zoonotic cutaneous leishmaniasis as a case system. *American Journal of Tropical Medicine and Hygiene* 97: 575–576.
121. Wood CL, McInturff A, Young HS, Kim D, Lafferty KD (2017) Human infectious disease burdens decrease with urbanization but not with biodiversity. *Philosophical Transactions of the Royal Society B: Biological Sciences* 372: 20160122.
122. Wu T, Perrings C, Kinzig A, Collins JP, Minteer BA, Daszak P (2017) Economic growth, urbanization, globalization, and the risks of emerging infectious diseases in China: A review. *Ambio* 46: 18–29.
123. Wu T, Perrings C (2017) Conservation, development and the management of infectious disease: avian influenza in China, 2004–2012. *Philosophical Transactions of the Royal Society B: Biological Sciences* 372: 20160126.
124. Xie Y, Hoberg EP, Yang Z, Urban JF, Yang G (2017) Ancylostoma ailuropodae n. sp. (Nematoda: Ancylostomatidae), a new hookworm parasite isolated from wild giant pandas in Southwest China. *Parasites & Vectors* 10: 277.
125. Young HS, McCauley DJ, Dirzo R, Nunn CL, Campana MG, Agwanda B et al. (2017) Interacting effects of land use and climate on rodent-borne pathogens in central Kenya. *Philosophical Transactions of the Royal Society B: Biological Sciences* 372: 20160116.
126. Zhang G, Xiao X, Biradar CM, Dong J, Qin Y, Menarguez MA et al. (2017) Spatiotemporal patterns of paddy rice croplands in China and India from 2000 to 2015. *Science of The Total Environment* 579: 82–92.
127. Brant HL, Ewers RM, Vythilingam I, Drakeley C, Benedick S, Mumford JD (2016) Vertical stratification of adult mosquitoes (Diptera: Culicidae) within a tropical rainforest in Sabah, Malaysia. *Malaria Journal* 15: 370.
128. Brierley L, Vonhof MJ, Olival KJ, Daszak P, Jones KE (2016) Quantifying Global Drivers of Zoonotic Bat Viruses: A Process-Based Perspective. *The American Naturalist* 187: E53–E64.
129. de Arruda MM, Figueiredo FB, Marcelino AP, Barbosa JR, Werneck GL, Noronha EF, Romero GAS (2016) Sensitivity and specificity of parallel or serial serological testing for detection of canine Leishmania infection. *Memorias Do Instituto Oswaldo Cruz* 111: 168–173.
130. De S, Debnath B (2016) Prevalence of health hazards associated with solid waste disposal - a case study of Kolkata, India. *Procedia Environmental Sciences* 35: 201–208.
131. Foley J, Serieys LEK, Stephenson N, Riley S, Foley C, Jennings M et al. (2016) A synthetic review of notoedres species mites and mange. *Parasitology* 143: 1847–1861.
132. Gordon CA, McManus DP, Jones MK, Gray DJ, Gobert GN (2016) The Increase of Exotic Zoonotic Helminth Infections: The Impact of Urbanization, Climate Change and Globalization. In: Rollinson D, Stothard JR (eds) *Advances in Parasitology, Vol 91*, Advances in Parasitology, 311-+.
133. Gossner C, Danielson N, Gervelmeyer A, Berthe F, Faye B, Aaslav KK et al. (2016) Human-Dromedary Camel Interactions and the Risk of Acquiring Zoonotic Middle East Respiratory Syndrome Coronavirus Infection. *Zoonoses and Public Health* 63: 1–9.
134. Gryseels S, Goüy de Bellocq J, Makundi R, Vanmechelen K, Broeckhove J, Mazoch V et al. (2016) Genetic distinction between contiguous urban and rural multimammate mice in Tanzania despite gene flow. *Journal of Evolutionary Biology* 29: 1952–1967.
135. Gulachenski A, Ghersi BM, Lesen AE, Blum MJ (2016) Abandonment, Ecological Assembly and Public Health Risks in Counter-Urbanizing Cities. *Sustainability* 8: 491.
136. Hancke D, Suarez O V (2016) Infection levels of the cestode Hymenolepis diminuta in rat populations from Buenos Aires, Argentina. *Journal of Helminthology* 90: 199–205.
137. Irga PJ, Armstrong B, King WL, Burchett M, Torpy FR (2016) Correspondence Between Urban Bird Roosts and the Presence of Aerosolised Fungal Pathogens. *Mycopathologia* 181: 689–699.
138. Lau CL (2016) Human Leptospirosis in Oceania. *Neglected Tropical Diseases*: 177–192.
139. Lau CL, Watson CH, Lowry JH, David MC, Craig SB, Wynwood SJ, Kama M, Nilles EJ (2016) Human Leptospirosis Infection in Fiji: An Eco-epidemiological Approach to Identifying Risk Factors and Environmental Drivers for Transmission. *PLOS Neglected Tropical Diseases* 10: e0004405.
140. Leo SST, Gonzalez A, Millien V (2016) Multi-taxa integrated landscape genetics for zoonotic infectious diseases: deciphering variables influencing disease emergence. *Genome* 59: 349–361.
141. Millan J, Proboste T, de Mera IGF, Chirife AD, de la Fuente J, Altet L (2016) Molecular detection of vector-borne pathogens in wild and domestic carnivores and their ticks at the human-wildlife interface. *Ticks and Tick-Borne Diseases* 7: 284–290.
142. Moyes CL, Shearer FM, Huang Z, Wiebe A, Gibson HS, Nijman V et al. (2016) Predicting the geographical distributions of the macaque hosts and mosquito vectors of Plasmodium knowlesi malaria in forested and non-forested areas. *Parasites & Vectors* 9: 242.
143. Parsons MH, Sarno RJ, Deutsch MA (2016) A Detailed Protocol to Enable Safe-Handling, Preemptive Detection, and Systematic Surveillance of Rat-Vectored Pathogens in the Urban Environment. *Frontiers in Public Health* 4: 132.
144. Pavlik I (2016) Volcanic soil erosion and degradation in Central American continental countries and impact on humans’ health. *Proceedings from international conference: Soil – the Non-renewable Environmental Resource,* 230–241.
145. Puckett EE, Park J, Combs M, Blum MJ, Bryant JE, Caccone A et al. (2016) Global population divergence and admixture of the brown rat (Rattus norvegicus). *Proceedings of the Royal Society B: Biological Sciences* 283: 20161762.
146. Ramirez JD, Hernandez C, Leon CM, Ayala MS, Florez C, Gonzalez C (2016) Taxonomy, diversity, temporal and geographical distribution of Cutaneous Leishmaniasis in Colombia: A retrospective study. *Scientific Reports* 6: 28266.
147. Rodriguez-Vivas RI, Apanaskevich DA, Ojeda-Chi MM, Trinidad-Martinez I, Reyes-Novelo E, Esteve-Gassent MD, de Leon AAP (2016) Ticks collected from humans, domestic animals, and wildlife in Yucatan, Mexico. *Veterinary Parasitology* 215: 106–113.
148. Santiago-Alarcon D, MacGregor-Fors I, Kuhnert K, Segelbacher G, Schaefer HM (2016) Avian haemosporidian parasites in an urban forest and their relationship to bird size and abundance. *Urban Ecosystems* 19: 331–346.
149. Schowalter TD (2016) Responses to Abiotic Conditions. *Insect Ecology*: 21–59.
150. Streicker DG, Allgeier JE (2016) Foraging choices of vampire bats in diverse landscapes: potential implications for land-use change and disease transmission. *Journal of Applied Ecology* 53: 1280–1288.
151. Streicker DG, Winternitz JC, Satterfield DA, Condori-Condori RE, Broos A, Tello C et al. (2016) Host–pathogen evolutionary signatures reveal dynamics and future invasions of vampire bat rabies. *Proceedings of the National Academy of Sciences* 113: 10926–10931.
152. Tilston-Lunel NL, Acrani GO, Randall RE, Elliott RM (2016) Generation of recombinant Oropouche viruses lacking the nonstructural protein NSm or NSs. *Journal of Virology* 90: 2616–2627.
153. Wang JZ, Sun QZ, Shao ZJ, Dou XF, Wang QY, Cui BY, Kan B, Xu JG (2016) Infectious Diseases and Urbanization. In: Li M, Wu YF (eds) *Urbanisation and Public Health in China*, 103-124. Imperial Coll Press, London, England.
154. Zanella JRC (2016) Emerging and reemerging zoonoses and their importance for animal health and production. *Pesquisa Agropecuaria Brasileira* 51: 510–519.
155. Abella-Medrano CA, Ibáñez-Bernal S, MacGregor-Fors I, Santiago-Alarcon D (2015) Spatiotemporal variation of mosquito diversity (Diptera: Culicidae) at places with different land-use types within a neotropical montane cloud forest matrix. *Parasites & Vectors* 8: 487.
156. Baily JL, Meric G, Bayliss S, Foster G, Moss SE, Watson E et al. (2015) Evidence of land-sea transfer of the zoonotic pathogen Campylobacter to a wildlife marine sentinel species. *Molecular Ecology* 24: 208–221.
157. Ben-Shimol S, Sagi O, Codish S, Novack V, Barrett C, Fruchtman Y, Berkowitz A, Shemer-Avni Y, Greenberg D (2015) Dramatic increase in laboratory-diagnosed human cutaneous leishmaniasis cases in southern Israel, 2007–2013. *Infectious Diseases* 47: 161–167.
158. Brashear WA, Ammerman LK, Dowler RC (2015) Short-distance dispersal and lack of genetic structure in an urban striped skunk population. *Journal of Mammalogy* 96: 72–80.
159. Brites J, Brasil J, Duarte KMR (2015) Epidemiological surveillance of capybaras and ticks on warning area for Brazilian spotted fever. *Veterinary World* 8: 1143–1149.
160. Campbell LP, Finley AO, Benbow ME, Gronseth J, Small P, Johnson RC et al. (2015) Spatial Analysis of Anthropogenic Landscape Disturbance and Buruli Ulcer Disease in Benin. *PLOS Neglected Tropical Diseases* 9: e0004123.
161. Chen J, Zhao R (2015) A Research on impacts of demographic factors on zoonosis along with the urbanization in China. *Proceedings of the 11th Euro-Asia Conference on Environment and CSR: Tourism, Society and Education Session (Part I),* 318–323.
162. Chomel B (2015) Lyme disease. *Revue Scientifique et Technique de l’OIE* 34: 569–576.
163. Cibot M, Guillot J, Lafosse S, Bon C, Seguya A, Krief S (2015) Nodular worm infections in wild non-human primates and humans living in the Sebitoli area (Kibale National Park, Uganda): do high spatial proximity favor zoonotic transmission? *Plos Neglected Tropical Diseases* 9: e0004133.
164. Costa F, Hagan JE, Calcagno J, Kane M, Torgerson P, Martinez-Silveira MS, Stein C, Abela-Ridder B, Ko AI (2015) Global morbidity and mortality of leptospirosis: a systematic review. *PLOS Neglected Tropical Diseases* 9: e0003898.
165. de Noya BA, González ON (2015) An ecological overview on the factors that drives to Trypanosoma cruzi oral transmission. *Acta Tropica* 151: 94–102.
166. de Oliveira AC, Figueiredo FB, Silva VL, Santos FN, de Souza MB, Madeira MD, Abrantes TR, Perisse ARS (2015) Canine visceral Leishmaniasis case investigation in the Jacare region on Niteroi, Rio de Janeiro, Brazil. *Revista Do Instituto De Medicina Tropical De Sao Paulo* 57: 325–332.
167. Debenham JJ, Atencia R, Midtgaard F, Robertson LJ (2015) Occurrence of Giardia and Cryptosporidiumin captive chimpanzees (Pan troglodytes), mandrills (Mandrillus sphinx) and wild Zanzibar red colobus monkeys (Procolobus kirkii). *Journal of Medical Primatology* 44: 60–65.
168. Dobigny G, Garba M, Tatard C, Loiseau A, Galan M, Kadaouré I, Rossi J-P, Picardeau M, Bertherat E (2015) Urban market gardening and rodent-borne pathogenic Leptospira in arid zones: a case study in Niamey, Niger. *PLOS Neglected Tropical Diseases* 9: e0004097.
169. European Food Safety Authority EF (2015) Drivers for occasional spillover event of Ebola virus. *Efsa Journal* 13: 4161.
170. Gadisa E, Tsegaw T, Abera A, Elnaiem D, den Boer M, Aseffa A, Jorge A (2015) Eco-epidemiology of visceral leishmaniasis in Ethiopia. *Parasites & Vectors* 8: 381.
171. Gilbert M, Conchedda G, Van Boeckel TP, Cinardi G, Linard C, Nicolas G et al. (2015) Income disparities and the global distribution of intensively farmed chicken and pigs. *PLOS ONE* 10: e0133381.
172. Guimaraes AGF, Alves GBM, Pessoa AD, da Silva NJ (2015) Spatial analysis of visceral leishmaniasis in the municipality of Rondonopolis, in the Brazilian State of Mato Grosso, from 2003 to 2012: human, canine and vector distribution in areas of disease transmission. *Revista Da Sociedade Brasileira De Medicina Tropical* 48: 291–300.
173. Jankielsohn A (2015) The hidden cost of eating meat in South Africa: what every responsible consumer should know. *Journal of Agricultural and Environmental Ethics* 28: 1145–1157.
174. Lal A, Lill AWT, McIntyre M, Hales S, Baker MG, French NP (2015) Environmental change and enteric zoonoses in New Zealand: a systematic review of the evidence. *Australian and New Zealand Journal of Public Health* 39: 63–68.
175. Lau CL, Skelly C, Dohnt M, Smythe LD (2015) The emergence of Leptospira borgpetersenii serovar Arborea in Queensland, Australia, 2001 to 2013. *BMC Infectious Diseases* 15: 230.
176. Liyanaarachchi DR, Rajakaruna RS, Dikkumbura AW, Rajapakse R (2015) Ticks infesting wild and domestic animals and humans of Sri Lanka with new host records. *Acta Tropica* 142: 64–70.
177. Loh EH, Zambrana-Torrelio C, Olival KJ, Bogich TL, Johnson CK, Mazet JAK, Karesh W, Daszak P (2015) Targeting transmission pathways for emerging zoonotic disease surveillance and control. *Vector-Borne and Zoonotic Diseases* 15: 432–437.
178. Mackenstedt U, Jenkins D, Romig T (2015) The role of wildlife in the transmission of parasitic zoonoses in peri-urban and urban areas. *International Journal for Parasitology: Parasites and Wildlife* 4: 71–79.
179. Metelka J, Robertson C, Stephen C (2015) Japanese encephalitis: estimating future trends in Asia. *AIMS Public Health* 2: 601–615.
180. Morand S, Bordes F, Blasdell K, Pilosof S, Cornu JF, Chaisiri K et al. (2015) Assessing the distribution of disease-bearing rodents in human-modified tropical landscapes. *Journal of Applied Ecology* 52: 784–794.
181. Okello AL, Burniston S, Conlan J V, Inthavong P, Khamlome B, Welburn SC, Gilbert J, Allen J, Blacksell SD (2015) Prevalence of endemic pig-associated zoonoses in Southeast Asia: A review of findings from the Lao People’s Democratic Republic. *American Journal of Tropical Medicine and Hygiene* 92: 1059–1066.
182. Pizzi H, Tomas A, Ferrero M, Fernandez G, Furey F, Pizzi R, Herrero M, Dib M (2015) The relentless progress of Leishmaniasis: report of the first autochthonous case in the province of Cordoba. *Revista De Salud Publica-Cordoba* 19: 15–23.
183. Zhan P, Li D, Wang C, Sun J, Geng C, Xiong Z, Seyedmousavi S, Liu W, de Hoog GS (2015) Epidemiological changes in tinea capitis over the sixty years of economic growth in China. *Medical Mycology* 53: 691–698.
184. Al-Sabi M, Halasa T, Kapel C (2014) Infections with cardiopulmonary and intestinal helminths and sarcoptic mange in red foxes from two different localities in Denmark. *Acta Parasitologica* 59: 98-107.
185. Berger R, Wasserberg G, Warburg A, Orshan L, Kotler BP (2014) Zoonotic disease in a peripheral population: persistence and transmission of Leishmania major in a putative sink-source system in the Negev Highlands, Israel. *Vector-Borne and Zoonotic Diseases* 14: 592–600.
186. Carmena D, Cardona GA (2014) Echinococcosis in wild carnivorous species: Epidemiology, genotypic diversity, and implications for veterinary public health. *Veterinary Parasitology* 202: 69–94.
187. Carrique-Mas JJ, Bryant JE, Cuong N V, Hoang NVM, Campbell J, Hoang N V et al. (2014) An epidemiological investigation of Campylobacter in pig and poultry farms in the Mekong delta of Vietnam. *Epidemiology and Infection* 142: 1425–1436.
188. Castro JG, Freire ML, Campos SPS, Scopel KKG, Porrozzi R, Da Silva ED et al. (2014) Evidence of Leishmania (Leishmania) infantum infection in dogs from Juiz de Fora, Minas Gerais State, Brazil, based on immunochromatographic dual-path platform (DPP®) and PCR assays. *Revista Do Instituto De Medicina Tropical De Sao Paulo* 56: 225–229.
189. Ducrotoy MJ, Bertu WJ, Ocholi RA, Gusi AM, Bryssinckx W, Welburn S, Moriyón I (2014) Brucellosis as an emerging threat in developing economies: lessons from Nigeria. *PLoS Neglected Tropical Diseases* 8: e3008.
190. Dupouey J, Faucher B, Edouard S, Richet H, Kodjo A, Drancourt M, Davoust B (2014) Human leptospirosis: An emerging risk in Europe? *Comparative Immunology, Microbiology and Infectious Diseases* 37: 77–83.
191. Estrada-Pena A, Ostfeld RS, Peterson AT, Poulin R, de la Fuente J (2014) Effects of environmental change on zoonotic disease risk: an ecological primer. *Trends in Parasitology* 30: 205–214.
192. Faraji A, Egizi A, Fonseca DM, Unlu I, Crepeau T, Healy SP, Gaugler R (2014) Comparative host feeding patterns of the Asian tiger mosquito, Aedes albopictus, in urban and suburban Northeastern USA and implications for disease transmission. *PLoS Neglected Tropical Diseases* 8: e3037.
193. Feliciangeli M (2014) Leishmaniasis in Venezuela: Current status, actions and prospects for vector control in the context of a multi-sectorial control program. *Boletin De Malariologia Y Salud Ambiental* 54: 1–7.
194. Gottdenker NL, Streicker DG, Faust CL, Carroll CR (2014) Anthropogenic land use change and infectious diseases: a review of the evidence. *EcoHealth* 11: 619–632.
195. Hernandez C, Alvarez C, Gonzalez C, Ayala MS, Leon CM, Ramirez JD (2014) Identification of six New World Leishmania species through the implementation of a High-Resolution Melting (HRM) genotyping assay. *Parasites & Vectors* 7: 502.
196. Iveson JB, Bradshaw SD, How RA, Smith DW (2014) Human migration is important in the international spread of exotic Salmonella serovars in animal and human populations. *Epidemiology and Infection* 142: 2281–2296.
197. Kock R (2014) Drivers of disease emergence and spread: Is wildlife to blame? *Onderstepoort J Vet Res* 81.
198. Lima VS, Jansen AM, Messenger LA, Miles MA, Llewellyn MS (2014) Wild Trypanosoma cruzi I genetic diversity in Brazil suggests admixture and disturbance in parasite populations from the Atlantic Forest region. *Parasites & Vectors* 7: 263.
199. Liu Q, Cao L, Zhu X-Q (2014) Major emerging and re-emerging zoonoses in China: a matter of global health and socioeconomic development for 1.3 billion. *International Journal of Infectious Diseases* 25: 65–72.
200. Marston CG, Danson FM, Armitage RP, Giraudoux P, Pleydell DRJ, Wang Q, Qui J, Craig PS (2014) A random forest approach for predicting the presence of Echinococcus multilocularis intermediate host Ochotona spp. presence in relation to landscape characteristics in western China. *Applied Geography* 55: 176–183
201. .Mehndiratta P, Bhalla P (2014) Use of antibiotics in animal agriculture & emergence of methicillin-resistant Staphylococcus aureus (MRSA) clones: Need to assess the impact on public health. *Indian Journal of Medical Research* 140: 339–344.
202. Montenegro H, Rodrigues AM, Dias MAG, da Silva EA, Bernardi F, de Camargo ZP (2014) Feline sporotrichosis due to Sporothrix brasiliensis: an emerging animal infection in São Paulo, Brazil. *BMC Veterinary Research* 10: 269.
203. NABARRO D, WANNOUS C (2014) The potential contribution of livestock to food and nutrition security: the application of the One Health approach in livestock policy and practice. *Revue Scientifique et Technique de l’OIE* 33: 475–485.
204. Oliveira IBB, Batista HL, Peluzio JM, Pfrimer IAH, Rodrigues FM, Carmo Filho JR do (2014) Epidemiological and environmental aspects of visceral leishmaniasis in children under 15 years of age between 2007 and 2012 in the City of Araguaína, State of Tocantins, Brazil. *Revista da Sociedade Brasileira de Medicina Tropical* 47: 476–482.
205. Paige SB, Frost SDW, Gibson MA, Jones JH, Shankar A, Switzer WM, Ting N, Goldberg TL (2014) Beyond Bushmeat: Animal Contact, Injury, and Zoonotic Disease Risk in Western Uganda. *Ecohealth* 11: 534–543.
206. Pernet O, Schneider BS, Beaty SM, LeBreton M, Yun TE, Park A et al. (2014) Evidence for henipavirus spillover into human populations in Africa. *Nature Communications* 5: 5342.
207. Plumer L, Davison J, Saarma U (2014) Rapid urbanization of red foxes in Estonia: distribution, behaviour, attacks on domestic animals, and health-risks related to zoonotic diseases. *PLoS ONE* 9: e115124.
208. Rubio A V, Avila-Flores R, Suzan G (2014) Responses of small mammals to habitat fragmentation: epidemiological considerations for rodent-borne Hantaviruses in the Americas. *Ecohealth* 11: 526–533.
209. Ta TH, Hisam S, Lanza M, Jiram AI, Ismail N, Rubio JM (2014) First case of a naturally acquired human infection with Plasmodium cynomolgi. *Malaria Journal* 13: 68.
210. Umhang G, Comte S, Raton V, Hormaz V, Boucher J-M, Favier S, Combes B, Boué F (2014) Echinococcus multilocularis infections in dogs from urban and peri-urban areas in France. *Parasitology Research* 113: 2219–2222.
211. Wang Q, Huang Y, Huang L, Yu W, He W, Zhong B et al. (2014) Review of risk factors for human echinococcosis prevalence on the Qinghai-Tibet Plateau, China: a prospective for control options. *Infectious Diseases of Poverty* 3: 3.
212. Witchell TD, Eshghi A, Nally JE, Hof R, Boulanger MJ, Wunder EA, Ko AI, Haake DA, Cameron CE (2014) Post-translational modification of LipL32 during Leptospira interrogans infection. *Plos Neglected Tropical Diseases* 8: e3280.
213. Wood CL, Lafferty KD, DeLeo G, Young HS, Hudson PJ, Kuris AM (2014) Does biodiversity protect humans against infectious disease? *Ecology* 95: 817–832.
214. Atkinson JAM, Gray DJ, Clements ACA, Barnes TS, McManus DP, Yang YR (2013) Environmental changes impacting Echinococcus transmission: research to support predictive surveillance and control. *Global Change Biology* 19: 677–688.
215. Barrios JM, Verstraeten WW, Maes P, Aerts JM, Farifteh J, Coppin P (2013) Relating land cover and spatial distribution of nephropathia epidemica and Lyme borreliosis in Belgium. *International Journal of Environmental Health Research* 23: 132–154.
216. Bayry J (2013) Emerging viral diseases of livestock in the developing world. *Indian Journal of Virology* 24: 291–294.
217. Beasley JC, Eagan TS, Page LK, Hennessy CA, Rhodes OE (2013) Baylisascaris procyonis infection in white-footed mice: predicting patterns of infection from landscape habitat attributes. *Journal of Parasitology* 99: 743–747.
218. Beasley JC, Olson ZH, Beatty WS, Dharmarajan G, Rhodes OE (2013) Effects of culling on mesopredator population dynamics. *PLoS ONE* 8: e58982.
219. Carmena D, Cardona GA (2013) Canine echinococcosis: Global epidemiology and genotypic diversity. *Acta Tropica* 128: 441–460.
220. Chen J, Zhao R (2013) A Research on the Environmental Pollution Resulting from Pets in Chinese Cities. *Proceedings of the 9^th^ Euro-Asia Conference on Environment and CSR: tourism, Society and Education Session (PT I).* Pages 19–23.
221. Cohen TM, King R, Dolev A, Boldo A, Lichter-Peled A, Bar-Gal GK (2013) Genetic characterization of populations of the golden jackal and the red fox in Israel. *Conservation Genetics* 14: 55–63.
222. Coura-Vital W, Reis AB, Fausto MA, Leal GG de A, Marques MJ, Veloso VM, Carneiro M (2013) Risk factors for seroconversion by Leishmania infantum in a cohort of dogs from an endemic area of Brazil. *PLoS ONE* 8: e71833.
223. de Freitas J, Sampaio A, Santos G, Lima A, Nunes-Pinheiro D (2013) Analysis of seasonality, tendencies and correlations in human and canine visceral leishmaniasis. *Acta Scientiae Veterinariae* 41: 1151.
224. Faccini-Martinez AA, Sotomayor HA (2013) Historical review of the plague in South America: a little-known disease in Colombia. *Biomedica* 33: 8–27.
225. Herrero M, Grace D, Njuki J, Johnson N, Enahoro D, Silvestri S, Rufino MC (2013) The roles of livestock in developing countries. *Animal* 7: 3–18.
226. Jones BA, Grace D, Kock R, Alonso S, Rushton J, Said MY et al. (2013) Zoonosis emergence linked to agricultural intensification and environmental change. *Proceedings of the National Academy of Sciences* 110: 8399–8404.
227. Kimman T, Hoek M, de Jong MCM (2013) Assessing and controlling health risks from animal husbandry. *NJAS - Wageningen Journal of Life Sciences* 66: 7–14.
228. Kooriyama T, Okamoto M, Yoshida T, Nishida T, Tsubota T, Saito A et al. (2013) Epidemiological study of zoonoses derived from humans in captive chimpanzees. *Primates* 54: 89–98.
229. Lindahl JF, Ståhl K, Chirico J, Boqvist S, Thu HTV, Magnusson U (2013) Circulation of Japanese Encephalitis virus in pigs and mosquito vectors within Can Tho City, Vietnam. *PLoS Neglected Tropical Diseases* 7: e2153.
230. Marzetti S, Carranza C, Roncallo M, Escobar GI, Lucero NE (2013) Recent trends in human Brucella canis infection. *Comparative Immunology, Microbiology and Infectious Diseases* 36: 55–61.
231. Mather S, Scott S, Temperton N, Wright E, King B, Daly J (2013) Current progress with serological assays for exotic emerging/re-emerging viruses. *Future Virology* 8: 745–755.
232. Olival KJ, Hoguet RL, Daszak P (2013) Linking the Historical Roots of Environmental Conservation with Human and Wildlife Health. *EcoHealth* 10: 224–227.
233. Pfaffle M, Littwin N, Muders S V, Petney TN (2013) The ecology of tick-borne diseases. *International Journal for Parasitology* 43: 1059–1077.
234. Picardeau M (2013) Diagnosis and epidemiology of leptospirosis. *Médecine et Maladies Infectieuses* 43: 1–9.
235. Salkeld DJ, Padgett KA, Jones JH (2013) A meta-analysis suggesting that the relationship between biodiversity and risk of zoonotic pathogen transmission is idiosyncratic. *Ecology Letters* 16: 679–686.
236. Shapiro JT, da Costa Lima Junior MS, Dorval MEC, de Oliveira França A, Cepa Matos M de F, Bordignon MO (2013) First record of Leishmania braziliensis presence detected in bats, Mato Grosso do Sul, southwest Brazil. *Acta Tropica* 128: 171–174.
237. Vidal SM, Fajardo PI, Gonzalez CG (2013) Veterinaty education in the area of food safety (including animal health, food pathogens and surveillance of foodborne diseases). *Revue Scientifique Et Technique-Office International Des Epizooties* 32: 425–431.
238. Weaver SC (2013) Urbanization and geographic expansion of zoonotic arboviral diseases: mechanisms and potential strategies for prevention. *Trends in Microbiology* 21: 360–363.
239. West KA, Heymann EW, Mueller B, Gillespie TR (2013) Patterns of Infection with Cryptosporidium sp and Giardia sp in Three Species of Free-Ranging Primates in the Peruvian Amazon. *International Journal of Primatology* 34: 939–945.
240. Wood CL, Lafferty KD (2013) Biodiversity and disease: a synthesis of ecological perspectives on Lyme disease transmission. *Trends in Ecology & Evolution* 28: 239–247.
241. Arizono N, Yamada M, Tegoshi T, Onishi K (2012) Molecular identification of oesophagostomum and trichuris eggs isolated from wild Japanese macaques. *Korean Journal of Parasitology* 50: 253–257.
242. Bevins SN, Carver S, Boydston EE, Lyren LM, Alldredge M, Logan KA et al. (2012) Three pathogens in sympatric populations of pumas, bobcats, and domestic cats: implications for infectious disease transmission. *Plos One* 7: e31403.
243. Bruzinskaite-Schmidhalter R, Sarkunas M, Malakauskas A, Mathis A, Torgerson PR, Deplazes P (2012) Helminths of red foxes (Vulpes vulpes) and raccoon dogs (Nyctereutes procyonoides) in Lithuania. *Parasitology* 139: 120–127.
244. Chen J (2012) A Research on common features and prevention of zoonosis in industrialization era. *Proceedings Of The 8th Euro-Asia Conference On Environment And Csr: Tourism, Mice, Hospitality Management And Education Session (Pt I),* 18–22.
245. da Silva MBT, Costa MMD, Torres CCD, Galhardo MCG, do Valle ACF, Magalhaes M, Sabroza PC, de Oliveira RM (2012) Urban sporotrichosis: a neglected epidemic in Rio de Janeiro, Brazil. *Cadernos De Saude Publica* 28: 1867–1880.
246. Delgado CA, French K (2012) Parasite-bird interactions in urban areas: Current evidence and emerging questions. *Landscape and Urban Planning* 105: 5–14.
247. Gray RR, Salemi M (2012) Integrative molecular phylogeography in the context of infectious diseases on the human-animal interface. *Parasitology* 139: 1939–1951.
248. Hamer SA, Lehrer E, Magle SB (2012) Wild birds as sentinels for multiple zoonotic pathogens along an urban to rural gradient in greater Chicago, Illinois. *Zoonoses and Public Health* 59: 355–364.
249. Howard CR, Fletcher NF (2012) Emerging virus diseases: can we ever expect the unexpected? *Emerging Microbes & Infections* 1: 1–9.
250. Khatchikian CE, Prusinski M, Stone M, Backenson PB, Wang IN, Levy MZ, Brisson D (2012) Geographical and environmental factors driving the increase in the Lyme disease vector Ixodes scapularis. *Ecosphere* 3: 85.
251. Kuchipudi S V, Dunham SP, Nelli R, White GA, Coward VJ, Slomka MJ, Brown IH, Chang KC (2012) Rapid death of duck cells infected with influenza: a potential mechanism for host resistance to H5N1. *Immunology and Cell Biology* 90: 116–123.
252. Li S, Hartemink N, Speybroeck N, Vanwambeke SO (2012) Consequences of landscape fragmentation on Lyme disease risk: a cellular automata approach. *PLos One* 7: e39612.
253. McFarlane R, Sleigh A, McMichael T (2012) Synanthropy of Wild Mammals as a Determinant of Emerging Infectious Diseases in the Asian-Australasian Region. *Ecohealth* 9: 24–35.
254. Pradier S, Lecollinet S, Leblond A (2012) West Nile virus epidemiology and factors triggering change in its distribution in Europe. *Revue Scientifique Et Technique-Office International Des Epizooties* 31: 829–844.
255. Pulliam JRC, Epstein JH, Dushoff J, Rahman SA, Bunning M, Jamaluddin AA et al. (2012) Agricultural intensification, priming for persistence and the emergence of Nipah virus: a lethal bat-borne zoonosis. *Journal of the Royal Society Interface* 9: 89–101.
256. Rabinowitz PM, Galusha D, Vegso S, Michalove J, Rinne S, Scotch M, Kane M (2012) Comparison of human and animal surveillance data for H5N1 influenza A in Egypt 2006–2011. *PLoS ONE* 7: e43851.
257. Salyer SJ, Gillespie TR, Rwego IB, Chapman CA, Goldberg TL (2012) Epidemiology and molecular relationships of Cryptosporidium spp. in people, primates, and livestock from Western Uganda. *PLoS Neglected Tropical Diseases* 6: e1597.
258. Sprague LD, Al-Dahouk S, Neubauer H (2012) A review on camel brucellosis: a zoonosis sustained by ignorance and indifference. *Pathogens and Global Health* 106: 144–149.
259. Swei A, Briggs CJ, Lane RS, Ostfeld RS (2012) Impacts of an introduced forest pathogen on the risk of lyme disease in California. *Vector-Borne and Zoonotic Diseases* 12: 623–632.
260. Thompson M, Mykytczuk N, Gooderham K, Schulte-Hostedde A (2012) Prevalence of the bacterium Coxiella burnetii in wild rodents from a Canadian Natural Environment Park. *Zoonoses and Public Health* 59: 553–560.
261. Vescio FM, Busani L, Mughini-Gras L, Khoury C, Avellis L, Taseva E, Rezza G, Christova I (2012) Environmental correlates of crimean-congo haemorrhagic fever incidence in Bulgaria. *Bmc Public Health* 12: 1116.
262. Aplin KP, Suzuki H, Chinen AA, Chesser RT, ten Have J, Donnellan SC et al. (2011) Multiple geographic origins of commensalism and complex dispersal history of black rats. *PLoS ONE* 6: e26357.
263. Biehler D (2011) Embodied wildlife histories and the urban landscape. *Environmental History* 16: 445–450.
264. Campos-Krauer JM, Wisely SM (2011) Deforestation and cattle ranching drive rapid range expansion of capybara in the Gran Chaco ecosystem. *Global Change Biology* 17: 206–218.
265. Cascio A, Bosilkovski M, Rodriguez-Morales AJ, Pappas G (2011) The socio-ecology of zoonotic infections. *Clinical Microbiology and Infection* 17: 336–342.
266. Colwell DD, Dantas-Torres F, Otranto D (2011) Vector-borne parasitic zoonoses: Emerging scenarios and new perspectives. *Veterinary Parasitology* 182: 14–21.
267. Harhay MO, Olliaro PL, Costa DL, Costa CHN (2011) Urban parasitology: visceral leishmaniasis in Brazil. *Trends in Parasitology* 27: 403–409.
268. Jenkins EJ, Schurer JM, Gesy KM (2011) Old problems on a new playing field: Helminth zoonoses transmitted among dogs, wildlife, and people in a changing northern climate. *Veterinary Parasitology* 182: 54–69.
269. Kays R, Tilak S, Crofoot M, Fountain T, Obando D, Ortega A et al. (2011) Tracking animal location and activity with an automated radio telemetry system in a tropical rainforest. *The Computer Journal* 54: 1931–1948.
270. Krief S, Krief JM, Kasenene J, Sevenet T, Hladik CM, Snounou G, Guillot J (2011) Great apes: who are they? Are they able to self-medicate? *Bulletin De L Academie Nationale De Medecine* 195: 1927–1935.
271. Lee K-S, Divis PCS, Zakaria SK, Matusop A, Julin RA, Conway DJ, Cox-Singh J, Singh B (2011) Plasmodium knowlesi: Reservoir hosts and tracking the emergence in humans and macaques. *PLoS Pathogens* 7: e1002015.
272. Santana KDO, Bavia ME, Lima AD, Guimaraes ICS, Soares ES, Silva MMN, Mendonca J, Martin MD (2011) Spatial distribution of triatomines (Reduviidae: Triatominae) in urban areas of the city of Salvador, Bahia, Brazil. *Geospatial Health* 5: 199–203.
273. Santos-Lopez G, Vallejo-Ruiz V, Rendon-Gonzalez L, Reyes-Leyva J (2011) Determining factors in the appearance and reappearance of viral infections. *Interciencia* 36: 22–30.
274. Appuhn K (2010) Ecologies of beef: eighteenth-century epizootics and the environmental history of early modern Europe. *Environmental History* 15: 268–287.
275. Chaves LF, Harrington LC, Keogh CL, Nguyen AM, Kitron UD (2010) Blood feeding patterns of mosquitoes: random or structured? *Frontiers in Zoology* 7: 3.
276. Coura JR, Borges-Pereira J (2010) Chagas disease: 100 years after its discovery. A systemic review. *Acta Tropica* 115: 5–13.
277. Cruz I, Acosta L, Gutiérrez MN, Nieto J, Cañavate C, Deschutter J, Bornay-Llinares FJ (2010) A canine leishmaniasis pilot survey in an emerging focus of visceral leishmaniasis: Posadas (Misiones, Argentina). *BMC Infectious Diseases* 10: 342.
278. de Oliveira LCP, de Araujo RR, Alves CR, Mouta-Confort E, Lopez JA, de Mendonca-Lima FW (2010) Seroprevalence and risk factors for canine visceral leishmaniasis in the endemic area of Dias D’Avila, State of Bahia, Brazil. *Revista Da Sociedade Brasileira De Medicina Tropical* 43: 400–404.
279. Johnson PTJ, Townsend AR, Cleveland CC, Glibert PM, Howarth RW, McKenzie VJ, Rejmankova E, Ward MH (2010) Linking environmental nutrient enrichment and disease emergence in humans and wildlife. *Ecological Applications* 20: 16–29.
280. Johnston AR, Gillespie TR, Rwego IB, McLachlan TLT, Kent AD, Goldberg TL (2010) Molecular epidemiology of cross-species Giardia duodenalis transmission in Western Uganda. *PLos Neglected Tropical Diseases* 4: e683.
281. Lau CL, Smythe LD, Craig SB, Weinstein P (2010) Climate change, flooding, urbanisation and leptospirosis: fuelling the fire? *Transactions of the Royal Society of Tropical Medicine and Hygiene* 104: 631–638.
282. Mathews F (2010) Wild animal conservation and welfare in agricultural systems. *Animal Welfare* 19: 159–170.
283. Rey J, Lounibos L, Padmanabha H, Mosquera M (2010) Emergence of dengue fever in America: patterns, processes and prospects. *Interciencia* 35: 800–806.
284. Richomme C, Afonso E, Tolon V, Ducrot C, Halos L, Alliot A et al. (2010) Seroprevalence and factors associated with Toxoplasma gondii infection in wild boar (Sus scrofa) in a Mediterranean island. *Epidemiology and Infection* 138: 1257–1266.
285. Wayant NM, Maldonado D, Rojas de Arias A, Cousiño B, Goodin DG (2010) Correlation between normalized difference vegetation index and malaria in a subtropical rain forest undergoing rapid anthropogenic alteration. *Geospatial health* 4: 179.
286. Beja-Pereira A, Bricker B, Chen SY, Almendra C, White PJ, Luikart G (2009) DNA genotyping suggests that recent Brucellosis outbreaks in the Greater Yellowstone area originated from elk. *Journal of Wildlife Diseases* 45: 1174–1177.
287. Carver S, Spafford H, Storey A, Weinstein P (2009) Dryland salinity and the ecology of Ross River virus: the ecological underpinnings of the potential for transmission. *Vector-Borne and Zoonotic Diseases* 9: 611–622.
288. de Almeida A, Faria RP, Pimentel MFA, Dahroug MAA, Turbino N, Sousa VRF (2009) Seroepidemiological survey of canine leishmaniasis in endemic areas of Cuiaba, State of Mato Grosso. *Revista Da Sociedade Brasileira De Medicina Tropical* 42: 156–159.
289. Field HE (2009) Bats and Emerging Zoonoses: Henipaviruses and SARS. *Zoonoses and Public Health* 56: 278–284.
290. Gardner-Santana LC, Norris DE, Fornadel CM, Hinson ER, Klein SL, Glass GE (2009) Commensal ecology, urban landscapes, and their influence on the genetic characteristics of city-dwelling Norway rats (Rattus norvegicus). *Molecular Ecology* 18: 2766–2778.
291. Gubler DJ (2009) Vector-borne diseases. *Revue Scientifique et Technique de l’OIE* 28: 583–588.
292. Korenberg EI (2009) Recent epidemiology of tick-borne encephalitis: an effect of climate change? In: Maramorosch K, Shatkin AJ, Murphy FA (eds) *Advances in Virus Research, Vol 74*, Advances in Virus Research, 123–144.
293. Lallo MA, Pereira A, Araujo R, Favorito SE, Bertolla P, Bondan EF (2009) Occurrence of giardia, cryptosporidium and microsporidia in wild animals from a deforestation area in the state of Sao Paulo, Brazil. *Ciencia Rural* 39: 1465–1470.
294. Leendertz F (2009) Tropical rainforests as reservoirs for emerging zoonoses. *Berliner Und Munchener Tierarztliche Wochenschrift* 122: 482–483.
295. Mackenzie JS, Williams DT (2009) The zoonotic flaviviruses of Southern, South-Eastern and Eastern Asia, and Australasia: the potential for emergent viruses. *Zoonoses and Public Health* 56: 338–356.
296. Pereira A, Araujo RS, Favorito SE, Bertolla PB, Lallo MA (2009) Occurrence of Microsporidia in small wildlife mammals in the state of Sao Paulo, Brazil. *Arquivo Brasileiro De Medicina Veterinaria E Zootecnia* 61: 1474–1477.
297. Sakudo A, Ikuta K (2009) Prion protein functions and dysfunction in prion diseases. *Current Medicinal Chemistry* 16: 380–389.
298. Teichroeb JA, Kutz SJ, Parkar U, Thompson RCA, Sicotte P (2009) Ecology of the gastrointestinal parasites of Colobus vellerosus at Boabeng-Fiema, Ghana: possible anthropozoonotic transmission. *American Journal of Physical Anthropology* 140: 498–507.
299. Wild TF (2009) Henipaviruses: A new family of emerging Paramyxoviruses. *Pathologie Biologie* 57: 188–196.
300. Woodford MH (2009) Veterinary aspects of ecological monitoring: the natural history of emerging infectious diseases of humans, domestic animals and wildlife. *Tropical Animal Health and Production* 41: 1023–1033.
301. Ximenes M, Pinheiro MPG, Cavalcante KB, Silva VPM, Pontes NN, Queiroz P V et al. (2009) Urbanization of visceral leishmaniasis in Rio Grande do Norte, Brazil: biotic and abiotic interactions. *Revista Da Sociedade Brasileira De Medicina Tropical* 42: 138–140.
302. Black PF, Murray JG, Nunn MJ (2008) Managing animal disease risk in Australia: the impact of climate change. *Revue Scientifique Et Technique-Office International Des Epizooties* 27: 563–580.
303. Bradley CA, Gibbs SEJ, Altizer S (2008) Urban land use predicts West Nile virus exposure in songbirds. *Ecological Applications* 18: 1083–1092.
304. Cabello C, Cabello F (2008) Zoonoses with wildlife reservoirs: A threat to public health and the economy. *Revista Medica De Chile* 136: 385–393.
305. Diniz SA, Silva FL, Neta AVC, Bueno R, Guerra R, Abreu-Silva AL, Santos RL (2008) Animal reservoirs for visceral leishmaniasis in densely populated urban areas. *Journal of Infection in Developing Countries* 2: 24–33.
306. Fahrion AS, Staebler S, Deplazes P (2008) Patent Toxocara canis infections in previously exposed and in helminth-free dogs after infection with low numbers of embryonated eggs. *Veterinary Parasitology* 152: 108–115.
307. Gascon J, Albajar P, Canas E, Flores M, Gomez i Prat J, Herrera RN et al. (2008) Diagnosis, management and treatment of chronic Chagas’ heart disease in areas where Trypanosoma cruzi infection is not endemic. *Enfermedades Infecciosas Y Microbiologia Clinica* 26: 99–106.
308. Hofle U, Blanco JM, Crespo E, Naranjo V, Jimenez-Clavero MA, Sanchez A, la Fuente J, Gortazar C (2008) West Nile virus in the endangered Spanish imperial eagle. *Veterinary Microbiology* 129: 171–178.
309. Logiudice K, Duerr STK, Newhouse MJ, Schmidt KA, Killilea ME, Ostfeld RS (2008) Impact of host community composition on Lyme disease risk. *Ecology* 89: 2841–2849.
310. Mettenleiter T, Boehle W (2008) Infectious diseases in a changing environment. *Archives of Animal Breeding* 51: 49–56.
311. Sousa VRF, de Almeida A (2008) Co-infection between visceral leishmaniasis and monocitic ehrlichiosis in dogs from Cuiaba, Mato Grosso. *Acta Scientiae Veterinariae* 36: 113–117.
312. Swaddle JP, Calos SE (2008) Increased avian diversity is associated with lower incidence of human West Nile infection: observation of the dilution effect. *PLoS ONE* 3: e2488.
313. Tourre YM, Jarlan L, Lacaux J-P, Rotela CH, Lafaye M (2008) Spatio-temporal variability of NDVI–precipitation over southernmost South America: possible linkages between climate signals and epidemics. *Environmental Research Letters* 3: 44008.
314. Walker M, Wilcox B, Wong M (2008) Waterborne zoonoses and changes in hydrologic response due to watershed development. *Coastal Watershed Management*: 349–367.
315. Wimberly MC, Baer AD, Yabsley MJ (2008) Enhanced spatial models for predicting the geographic distributions of tick-borne pathogens. *International Journal of Health Geographics* 7: 15.
316. Daszak P, Epstein JH, Kilpatrick AM, Aguirre AA, Karesh WB, Cunningham AA (2007) Collaborative research approaches to the role of wildlife in zoonotic disease emergence. *Current Topics in Microbiology and Immunology*: 463–475.
317. Devendra C (2007) Perspectives on animal production systems in Asia. *Livestock Science* 106: 1–18.
318. Gascon J, Albajar P, Canas E, Flores M, Prat JGI, Herrera RN et al. (2007) Diagnosis, management and treatment of chronic Chagas’ heart disease in areas where Trypanosoma cruzi infection is not endemic. *Revista Espanola De Cardiologia* 60: 285–293.
319. Greger M (2007) The human/animal interface: emergence and resurgence of zoonotic infectious diseases. *Critical Reviews in Microbiology* 33: 243–299.
320. Linard C, Lamarque P, Heyman P, Ducoffre G, Luyasu V, Tersago K, Vanwambeke SO, Lambin EF (2007) Determinants of the geographic distribution of Puumala virus and Lyme borreliosis infections in Belgium. *International Journal of Health Geographics* 6: 15.
321. Manangan JS, Schweitzer SH, Nibbelink N, Yabsley MJ, Gibbs SEJ, Wimberly MC (2007) Habitat factors influencing distributions of Anaplasma phagocytophilum and Ehrlichia chaffeensis in the Mississippi alluvial valley. *Vector-Borne and Zoonotic Diseases* 7: 563–573.
322. Martinez S, Vanwambeke SO, Ready P (2007) *Linking changes in landscape composition and configuration with sandfly occurrence in southwest France*. In *2007 International Workshop on the Analysis of Multi-temporal Remote Sensing Images*. Pages 1-5.
323. Mendez JA, Parra E, Neira M, Rey GJ (2007) Detection of yellow fever virus by reverse transcriptase polymerase chain reaction in wild monkeys: a sensitive tool for epidemiologic surveillance. *Biomedica* 27: 461–467.
324. Parker S, Nuara A, Buller RML, Schultz DA (2007) Human monkeypox: an emerging zoonotic disease. *Future Microbiology* 2: 17–34.
325. Taku A, Bhat M, Dutta T, Chhabra R (2007) Viral diseases transmissible from non-human primates to man. *Indian Journal of Virology* 18: 47–56.
326. Gillespie TR (2006) Noninvasive assessment of gastrointestinal parasite infections in free-ranging primates. *International Journal of Primatology* 27: 1129–1143.
327. Giraudoux P, Pleydell D, Raoul F, Quéré J-P, Wang Q, Yang Y et al. (2006) Transmission ecology of Echinococcus multilocularis: What are the ranges of parasite stability among various host communities in China? *Parasitology International* 55: S237–S246.
328. Romig T, Thoma D, Weible A-K (2006) Echinococcus multilocularis – a zoonosis of anthropogenic environments? *Journal of Helminthology* 80: 207–212.
329. Rotureau B (2006) Ecology of the Leishmania species in the Guianan ecoregion complex. *The American Journal of Tropical Medicine and Hygiene* 74: 81–96.
330. Rotureau B (2006) Are New World leishmaniases becoming anthroponoses? *Medical Hypotheses* 67: 1235–1241.
331. Suzan G, Giermakowski JT, Marce E, Suzan-Azpiri H, Armien B, Yates TL (2006) Modeling hantavirus reservoir species dominance in high seroprevalence areas on the Azuero Peninsula of Panama. *American Journal of Tropical Medicine and Hygiene* 74: 1103–1110.
332. Duplantier J-M, Duchemin J-B, Chanteau S, Carniel E (2005) From the recent lessons of the Malagasy foci towards a global understanding of the factors involved in plague reemergence. *Veterinary Research* 36: 437–453.
333. Fischer C, Reperant LA, Weber JM, Hegglin D, Deplazes P (2005) Echinococcus multilocularis infections of rural, residential and urban foxes (Vulpes vulpes) in the canton of Geneva, Switzerland. *Parasite-Journal De La Societe Francaise De Parasitologie* 12: 339–346.
334. Sherikar AT, Waskar VS (2005) Emerging zoonoses and social-economic impact in India - A review. *Indian Journal of Animal Sciences* 75: 700–705.
335. Suzan G, Ceballos G (2005) The role of feral mammals on wildlife infectious disease prevalence in two nature reserves within Mexico City limits. *Journal of Zoo and Wildlife Medicine* 36: 479–484.
336. Wolfe ND, Daszak P, Kilpatrick AM, Burke DS (2005) Bushmeat hunting deforestation, and prediction of zoonoses emergence. *Emerging Infectious Diseases* 11: 1822–1827.
337. King LJ, Marano NN, Hughes JM (2004) New partnerships between animal health services and public health agencies. *Revue Scientifique et Technique de l’OIE* 23: 717–726.
338. Slingenbergh J, Gilbert M, Balogh KD, Wint W (2004) Ecological sources of zoonotic diseases. *Revue Scientifique et Technique de l’OIE* 23: 467–484.
339. Giraudoux P, Craig PS, Delattre P, Bao G, Bartholomot B, Harraga S, Quéré JP, Raoul F, Wang Y, Shi D, Vuitton, D.A (2003) Interactions between landscape changes and host communities can regulate Echinococcus multilocularis transmission. *Parasitology* 127: S119–S129.
340. Scarfe A (2003) State, regional, national, and international aquatic animal health policies: Focus for future aquaculture biosecurity. *Biosecurity in Aquaculture Production Systems: Exclusion of Pathogens and other Undesirables.* Pages 23–26.
341. Twiddy SS, Holmes EC, Rambaut A (2003) Inferring the rate and time-scale of dengue virus evolution. *Molecular Biology and Evolution* 20: 122–129.
342. Hensel A, Neubauer H (2002) Human pathogens associated with on-farm practices - Implications for control and surveillance strategies. *Food Safety Assurance in the Pre-Harvest Phase, Vol 1: Safety, Assurance and Veterinary Public Health*. Pages 125–139.
343. Gongal G, Rai J (2001) Human rabies in Nepal. *Rabies Control in Asia, 4th International Symposium on Rabies Control in Asia,* 231–237.
344. Patz JA (2001) Public health risk assessment linked to climatic and ecological change. *Human and Ecological Risk Assessment* 7: 1317-+.
345. Craig PS, Giraudoux P, Shi D, Bartholomot B, Barnish G, Delattre P et al. (2000) An epidemiological and ecological study of human alveolar echinococcosis transmission in south Gansu, China. *Acta Tropica* 77: 167–177.
346. Girard M (2000) Emerging infectious diseases. *M S-Medecine Sciences* 16: 883–891.
347. Patz JA, Graczyk TK, Geller N, Vittor AY (2000) Effects of environmental change on emerging parasitic diseases. *International Journal for Parasitology* 30: 1395–1405.
348. Gagneux P, Wills C, Gerloff U, Tautz D, Morin PA, Boesch C et al. (1999) Mitochondrial sequences show diverse evolutionary histories of African hominoids. *Proceedings of the National Academy of Sciences of the United States of America* 96: 5077–5082.
349. Chomel BB (1998) New emerging zoonoses: a challenge and an opportunity for the veterinary profession. *Comparative Immunology Microbiology and Infectious Diseases* 21: 1–14.
350. Priemer J, Jakob W (1998) Hydatid echinococcosis in a zoo-born dromedary from the Tierpark Berlin-Friedrichsfelde, Germany. *Berliner Und Munchener Tierarztliche Wochenschrift* 111: 100–103.
351. Pavlovic I, Kulisic Z, Milutinovic M (1997) The role of foxes (Vulpes vulpes L) in the epizootiology and epidemiology of nematode parasitic zoonoses. *Acta Veterinaria-Beograd* 47: 177–182.
352. Dhanda V, Das P, Lal R, Srinivasan R, Ramaiah K (1996) Spread of lymphatic filariasis, re-emergence of leishmaniasis & threat of babesiosis in India. *Indian Journal of Medical Research* 103: 46–54.
353. Meslin FX (1995) Zoonoses in the world – current and future trends. *Schweizerische Medizinische Wochenschrift* 125: 875–878.
354. Wijeyaratne PM, Arsenault LKJ, Murphy CJ (1994) Endemic disease and development: the leishmaniases. *Acta Tropica* 56: 349–364.
355. Mott K, Desjeux P, Moncayo A, Ranque P, Deraadt P (1990) Parasitic diseases and urban-development. *Bulletin of the World Health Organization* 68: 691–698.
356. Ahmed S, Davila JD, Allen A, Haklay M, Tacoli C, Fevre EM (2019) Does urbanization make emergence of zoonosis more likely? Evidence, myths and gaps. *Environment and Urbanization* 31: 443.
357. Ledger ML, Mitchell PD (2019) Tracing zoonotic parasite infections throughout human evolution. *International Journal of Osteoarchaeology* 1-12.
